# New insights in the control of antioxidants accumulation in tomato by transcriptomic analyses of genotypes exhibiting contrasting levels of fruit metabolites

**DOI:** 10.1186/s12864-019-5428-4

**Published:** 2019-01-15

**Authors:** Adriana Sacco, Assunta Raiola, Roberta Calafiore, Amalia Barone, Maria Manuela Rigano

**Affiliations:** Department of Agricultural Sciences, University of Naples Federico II, Portici, Naples, Italy

**Keywords:** *Solanum lycopersicum*, Ascorbic acid, Phenylpropanoids, RNA sequencing, WGCNA analyses, Transcription factor

## Abstract

**Background:**

Tomato is an economically important crop with fruits that are a significant source of bioactive compounds such as ascorbic acid and phenolics. Nowadays, the majority of the enzymes of the biosynthetic pathways and of the structural genes controlling the production and the accumulation of antioxidants in plants are known; however, the mechanisms that regulate the expression of these genes are yet to be investigated. Here, we analyzed the transcriptomic changes occurring during ripening in the fruits of two tomato cultivars (E1 and E115), characterized by a different accumulation of antioxidants, in order to identify candidate genes potentially involved in the biosynthesis of ascorbic acid and phenylpropanoids.

**Results:**

RNA sequencing analyses allowed identifying several structural and regulator genes putatively involved in ascorbate and phenylpropanoids biosynthesis in tomato fruits. Furthermore, transcription factors that may control antioxidants biosynthesis were identified through a weighted gene co-expression network analysis (WGCNA). Results obtained by RNA-seq and WGCNA analyses were further confirmed by RT-qPCR carried out at different ripening stages on ten cultivated tomato genotypes that accumulate different amount of bioactive compounds in the fruit. These analyses allowed us to identify one pectin methylesterase, which may affect the release of pectin-derived D-Galacturonic acid as metabolic precursor of ascorbate biosynthesis. Results reported in the present work allowed also identifying one L-ascorbate oxidase, which may favor the accumulation of reduced ascorbate in tomato fruits. Finally, the pivotal role of the enzymes chalcone synthases (CHS) in controlling the accumulation of phenolic compounds in cultivated tomato genotypes and the transcriptional control of the *CHS* genes exerted by Myb12 were confirmed.

**Conclusions:**

By using transcriptomic analyses, candidate genes encoding transcription factors and structural genes were identified that may be involved in the accumulation of ascorbic acid and phenylpropanoids in tomato fruits of cultivated genotypes. These analyses provided novel insights into the molecular mechanisms controlling antioxidants accumulation in ripening tomato fruits. The structural genes and regulators here identified could also be used as efficient genetic markers for selecting high antioxidants tomato cultivars.

**Electronic supplementary material:**

The online version of this article (10.1186/s12864-019-5428-4) contains supplementary material, which is available to authorized users.

## Background

In the last few years consumers are developing an increasing interest in vegetable crops, encouraged also by the health effects of the Mediterranean diet. Indeed, consumption of tomato fruits, fresh or processed, is associated with a reduced risk of cancer, inflammation and chronic non-communicable diseases (CNCD) including cardiovascular diseases (CVD) [1, 2]. These health effects are due to the presence in tomato fruits of bioactive substances such as vitamin C (ascorbic acid), polyphenols and carotenoids [3]. Polyphenolic compounds are associated with therapeutic roles in inflammatory diseases, neurodegenerative diseases, various type of cancers, and aging [2, 4]. Ascorbic acid (AsA), which cannot be synthesized by human body, shows significant ability as electron donor and potent antioxidant in human; it exerts a relevant role in protecting DNA from oxidant species induced damages and in the prevention of inflammation; it protects against oxidation of LDL (low-density lipoprotein) by different types of oxidative stress [4, 5]. In plants, polyphenolic compounds are secondary metabolites implicated in protection against damage from ultraviolet light, control of growth and developmental processes, pollination, and plant defense [6, 7]. Ascorbate can scavenge reactive oxygen species produced by photosynthesis and plays an important role in cell expansion, cell division, developmental processes and responses to stresses [8].

The general phenylpropanoids metabolism starts from phenylalanine and then, thanks to the activity of the enzymes PAL (phenylalanine ammonia lyase), C4H (cinnamate-4-hydroxylase) and 4CL (4-coumaroyl-CoA-ligase), the substrate p-coumaroyl CoA, which is the intermediated compound for the various branches of the phenylpropanoids pathway, is generated [9]. In the flavonoid pathway, the compound coumaroyl CoA is condensed with malonyl CoA in a reaction catalyzed by chalcone synthase (CHS) [10]. The different steps of the general phenylpropanoids biosynthetic pathway and for the biosynthesis of flavonoids, isoflavonoid, lignin, coumarins and other phenolics have been elucidated, and structural genes of the pathways have been isolated and characterized [10–12]. It has been demonstrated that plants produce ascorbic acid (AsA) through a number of biosynthetic pathways, and, recently, a bioinformatics approach has been used in order to reconstruct these pathways in tomato [13]. The prevalent AsA biosynthetic pathway, known also as the Smirnoff-Wheeler pathway, is the one that uses GDP-mannose and then proceed through L-galactose [14, 15]. However, it has been hypothesized the existence of a side branch of this pathway through GDP-gulose and the presence of alternative D-galacturonate and myo-inositol pathways for AsA biosynthesis [15, 16]. In the D-galacturonate pathway AsA could be produced either through the reduction of D-galacturonate resulting from pectin de-methylesterification and pectin degradation by pectin methylesterases (PMEs) and polygalacturonases, or from UDP-glucuronate epimerisation [14, 15]. Recycling of oxidized forms and AsA translocation across cellular compartments can also contribute to regulate ascorbate accumulation in plants [17–19].

The level of antioxidants in plants is highly influenced by different environmental conditions and also by the fruit developmental stage, indicating that multiple transcription factors (TFs) or regulators may act to control final antioxidants accumulation [20]. A number of transcription factors, such as the F-box AMR1, the HD-Zip I TF SlHZ24 and the TF SlDof22, have been shown to be involved in regulating ascorbic acid content; while the Myb transcription factors, such as Myb12, are known to regulate phenylpropanoids accumulation [20, 21]. Flavonoid biosynthesis is cooperatively regulated in plants by transcriptional regulators including Myb, bHLH (basic helix-loop-helix) and WD40 proteins that form a complex, called MBW, which activates transcription of structural genes of the biosynthetic pathways [7, 22]. Other regulatory factors may affect phenylpropanoids biosynthesis by binding to the MBW complex or by modulating the expression of structural genes [22]. Nevertheless, the transcription factors that modulate the expression of structural genes of the antioxidants biosynthetic pathways are still largely unknown [20]. The investigation and characterization of novel transcription factors and molecular mechanisms regulating antioxidants accumulation in tomato fruit during ripening would be extremely useful for plant research and breeding efforts aimed at improving this crop.

The development of RNA sequencing (RNA-seq) technology has provided plant researchers with a highly efficient and powerful tool that includes deep sequencing technologies to generate millions of short cDNA reads and that is therefore more efficient than traditional microarray analysis [23]. In the last few years RNA-seq studies have been carried out in different plants species including Arabidopsis, grape, maize, apple and also in tomato [20, 24]. In this last crop, RNA-seq has been used to investigate several mechanisms such as hormone-mediated fruit ripening and/or the accumulation of secondary metabolites [20]. In recent years, transcriptome analyses have been successfully carried out for the identification of candidate genes associated to antioxidants accumulation [20]. Here, we analyzed the transcriptomic changes occurring during ripening in the fruits of two tomato cultivars (E1 and E115) characterized by a different accumulation of antioxidants, in order to identify candidate genes potentially involved in the biosynthesis of ascorbic acid and phenylpropanoids. Based on the RNA sequencing dataset generated, we were able to identify several potential structural genes and transcription factors related to the biosynthesis of ascorbic acid and phenylpropanoids. In addition, RT-qPCR analyses on ten different cultivated tomato genotypes at different ripening stages were performed in order to confirm the involvement of the identified structural genes and transcription factors in the accumulation of antioxidants in tomato fruits. In this paper, a weighted gene co-expression network (WGCNA) analysis was also performed in order to identify other genes involved in antioxidants accumulation in tomato. WGCNA has been recently developed to more efficiently investigate transcriptomic analyses since it can capture the relationships of individual genes comprehensively, allowing to obtain information on both genes function and the mechanisms controlling the traits of interest [25]. This method has been recently used to dissect fruit anthocyanin and fruit acidity in apples, pollination in petunias and aporphine alkaloid biosynthesis in lotus [24, 25]. In this paper, WGCNA was used to predict the regulator genes involved in the biosynthesis of ascorbic acid and phenylpropanoids in tomato fruit.

## Methods

### Plant material

The tomato genotype E1 (Belmonte PBL01) is an Italian genotype used for fresh market. The genotype E115 (PI129882 from the US NPGS germplasm bank, [26]) was collected in Peru (South America). These two cultivated tomato genotypes were grown (three replicates *per* genotypes and 10 plants *per* replica) for four consecutive years (2013–2016) in an experimental open field located in Acerra (Lat. 40°56′50″ N Long. 14°22′21″ E, Naples, Italy) under standard agronomic practices. Eight tomato cultivated genotypes, (E14, E27, E43, E87, E102, E103, E109, E111) were grown in the same conditions in the years 2015–2016. During each trial season, fruits were harvested at three developmental stages: mature green (MG – 40 days post anthesis), breaker (BR – 45 days post anthesis) and mature red (MR – 55 days post anthesis). Sampled fruits were cut into pieces, frozen in liquid nitrogen and stored at − 80 °C for subsequent analyses. Information on the genotypes used in this study is available in Additional file 1. Photos and details on source and distribution of the genotypes used in this study are deposited on LabArchives (10.6070/H4TT4NXN [27]).

### Ascorbic acid quantification

Ascorbic acid (AsA) content was determined as reported by Stevens et al. [28] with slight modifications reported by Rigano et al. [2]. In brief, 500 mg of tomato frozen powder from fruits at different ripening stages were added to 300 μL of ice-cold 6% trichloroacetic acid (TCA). Samples were mixed and left on ice for 15 min, then centrifuged for 15 min at 25,000×g at 4 °C. Twenty μL of supernatant were transferred to a clean tube with 20 μl of 0.4 M phosphate buffer (pH 7.4) and 10 μl of double distilled (dd) H_2_O. Afterwards, 80 μl of reagent solution, prepared by mixing solution A [31% H_3_PO_4_, 4.6% (*w*/*v*) TCA and 0.6% (w/v) FeCl_3_] with solution B [4% 2,20-dipyridil (w/v) made in 70% ethanol] at a proportion of 2.75:1 (*v*/v), were added. The mixture was incubated at 37 °C for 40 min and measured at 525 nm by using a NanoPhotometerTM (Implen). Three separated biological replicates for each sample and three technical assays for each biological repetition were measured. Values were expressed as mg/100 g of fresh weight (FW).

### Phenylpropanoids quantification

Methanolic extracts were obtained by adding 70% methanol (30 mL) to 3 g of tomato frozen powder and the mixture was put in an ultrasonic bath for 60 min at 30 °C. The mixture was then centrifuged at 3500×*g* using a Rotina 420R Hettich 84 Zentrifugen centrifuge (Tuttlingen, Germany) for 10 min at 4 °C, and the supernatant was kept at − 20 °C until evaluation of total phenolic compounds and HPLC analysis.

Total phenolics were determined by the Folin–Ciocalteu assay [29], with modifications reported by Rigano et al. [2]. Briefly, Folin-Ciocalteu’s phenol reagent (62.5 μL) and dd H_2_O (250 μL) were added to a supernatant (62.5 μL) obtained from the hydrophilic extract. After 6 min, 7% Na_2_CO_3_ solution (625 μL), and dd H_2_O (500 μL) were added to the mixture, which was incubated for 90 min and the absorbance was read at 760 nm. Total phenolics content of tomato fruits was expressed as mg gallic acid equivalents (GAE)/100 g FW. Three biological replicates and three technical assays for each biological repetition were analyzed.

Twenty-five millilitres of methanolic extracts, obtained from the genotypes E1 and E115, were dried by a rotary evaporator (Buchi R-210, Milan, Italy) and dissolved in 70% methanol (500 μL) containing around 0.175 g of solid weight. The extract was passed through a 0.45 μm Millipore nylon filter (Merck Millipore, Bedford, MA, USA). Flavonoids and phenolic acids were identified and quantified by using a HPLC Spectra System SCM 1000 (Thermo Electron Corporation, San Jose, CA, USA) equipped with a Gemini column (3 μm C18, 110 A, 250 × 4.6 mm; Phenomenex, Torrance, CA, USA) and UV-visible detector (Shimadzu, Riverwood Drive, Columbia, MD) according to the procedure reported by Rigano et al. [2]. Chromatograms were recorded at 256 nm for rutin, quercetin and derivatives, 280 nm for naringenin, 330 nm for chlorogenic acid and derivatives, caffeic acid, kampferol-rutinoside, naringenin chalcone and derivatives. For quantification, integrated peak areas from the tested extracts were compared to the peak areas of known amounts of standard phenolic compounds. The results were expressed as mg/100 g FW.

### RNA-seq library construction and sequencing

RNA sequencing experiment was performed on 18 RNA samples obtained from the genotypes E1 and E115 (two genotypes *per* three biological replicates *per* three developmental stages). Total RNA was isolated from 3 g of tomato fruit powder by using TRIzol® RNA Isolation Reagents (Invitrogen, Carlsbad, CA, USA) according to the manufacturer’s instructions. The extracted RNA was then sent to the center Genomix4life (Università degli Studi di Salerno, Salerno, Italy) for quality check, libraries preparation, and sequencing. The samples were sequenced by using an Illumina HiSeq 2000 platform. A single-end tag sequencing strategy was chosen. After the raw reads were generated, adapter sequences and low quality read portions were trimmed using Trimmomatic program [30] while preserving the longest high quality part of a NGS read. The minimum length established was 25 bp and the quality score 35, which increases the quality and reliability of the analysis. Quality of the trimmed reads was ascertained by using the FastQC program [31]. The transcriptomic data supporting the results of this article are available in the NCBI Sequence Read Archive (SRA) under the accession number PRJNA390282 (http://ncbi.nlm.nih.gov.sra/) [32].

### Reads mapping and analysis

All cleaned reads were aligned against the *Solanum lycopersicum* reference genome sequence (version 2.50) [33] with TopHat (version 2.0.12), with a min-coverage-intron 10, max-coverage-intron 12,000, min-segment-intron 10, max-segment-intron 12,000 and b2-very-sensitive [34, 35]. The resulting alignment files were used as input for FeatureCounts (Subread package, version 1.4.5) together with the ITAG 2.40 annotation file to calculate gene expression values (raw read counts). The minimum mapping quality score used in FeatureCounts was 30. Only uniquely mapping reads were used for read counting. The overall quality of the experiment was evaluated by a PCA analysis, on the basis of the similarity between replicates. The similarity between replicates was evaluated by the calculation of Euclidean distance between the samples and by hierarchical clustering. The HTSFilter package was chosen for the removal of the not expressed genes and the ones showing too much variability. The ‘Trimmed Means of M-values’ (TMM) normalization strategy and a length of sequence of filtering thresholds = 25 were used. Once the consistency of the samples has been evaluated, and the lowly/variable-expressed genes have been discarded, differential expression analysis has been performed. The identification of the differentially expressed genes was performed with the package edgeR (version 3.6.8). In order to detect the differentially expressed genes, comparisons of the two tomato genotypes E1 and E115 at three different stages were performed. Differentially expressed genes (DEGs) were deemed significant based on the following criteria: genes were scored and the false discovery rates (FDRs) of the statistical test were less than 0.05.

### Co-expression network analysis

The gene co-expression network analyses were carried out using the R package WGCNA [36]. Before network construction the proper soft-thresholding power (β) was determined through the network topology analysis (sup_soft_power) and resulted equal to 18. The resulting adjacency matrix was then converted to a topological overlap (TO) matrix by the TOM similarity algorithm. The modules were obtained using the automatic network construction function block-wise Modules with default settings, except that the power is 20, TOMType is signed and mergeCutHeight is 0.10. The eigengene value was calculated for each module and used to test the association with each metabolite. The total connectivity and intramodular connectivity were calculated with weighted and Pearson correlations function.

### RT-qPCR analyses

The expression of candidate genes in tomato fruits of selected genotypes was verified by RT-qPCR amplification. Total RNA was isolated as described before and treated with RNase-free DNase (Invitrogen, Carlsbad, CA, USA Madison, WI, USA) according to the method reported by the manufacturer. Total RNA (1 μg) was treated by the Transcriptor High Fidelity cDNA Synthesis Kit (Roche, Mannheim, Germany) and 1 μL of cDNA diluited 1:5 was used for RT-qPCR analyses. In a final volume of 25 μL diluted cDNA was mixed with 12.5 μL SYBR Green PCR master mix (Applied Biosystems, Foster City, CA., U.S.A) and 5 pmol each of forward and reverse primers (Additional file 2). The reaction was carried out by using the 7900HT Fast-Real Time PCR System (Applied Biosystems) and the amplification program was performed according to the following steps: 2 min at 50 °C, 10 min at 95 °C, 0.15 min at 95 °C and 60 °C for 1 min for 40 cycles. The amplification program was followed by a thermal denaturing step (0.15 min at 95 °C, 0.15 min at 60 °C, 0.15 min at 95 °C). All reactions were run in triplicate for each biological replicates and as reference gene a housekeeping gene coding for the *elongation factor 1-α* (*Ef 1- α* – *Solyc06g005060*) was used [16]. The level of expression relative to the reference gene was calculated using the formula 2^-∆CT^, where ∆CT = (CT _RNA target_ - CT _reference RNA_) [37]. Comparison of RNA expression was based on a comparative CT method (∆∆CT) and the relative expression has been quantified and expressed according to log_2_RQ. RQ was calculated as 2^-∆∆CT^ and ∆∆CT = (CT _RNA target_ - CT _reference RNA_) - (CT _calibrator_ - CT _reference RNA_) [38, 39]. E1 was selected as calibrator. Quantitative results were expressed as the mean value ± SE.

### Statistical analyses

Differences of expression of candidate genes among samples in RT-qPCR analyses, and differences among analyzed genotypes in metabolic analyses were determined by using SPSS (Statistical Package for Social Sciences) Package 6, version 15.0 (SSPS Inc., Chicago, IL, USA). In RT-qPCR analyses significant different expression levels were determined by comparing the genotypes through a student’s t-test at a significance level of 0.05. In metabolic analyses, quantitative results were expressed as the mean value ± SD. Significant different metabolite levels were determined by comparing mean values through a factorial analysis of variance (ANOVA) with Duncan post hoc test at a significance level of 0.05.

## Results

### Metabolites content in the genotypes E1 and E115

Two tomato genotypes (E1 and E115) were selected from a population of 96 accessions previously grown and phenotyped for different quality traits in red ripe fruit, including the content of ascorbic acid (AsA) and total phenolics (Phe) [40]. According to that study, the two genotypes were classified as low-metabolites content (E1) and high-metabolites content (E115), respectively.

**Fig. 1:**
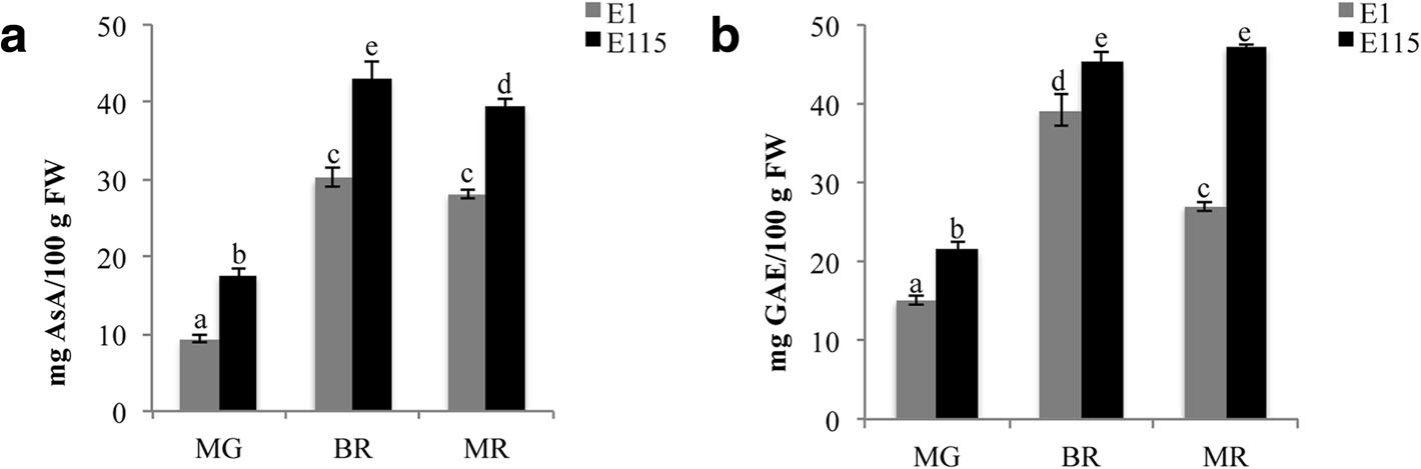
Content of antioxidants in tomato fruits. The content of ascorbic acid (**a**) and total phenolics (**b**) was calculated in fruits at three different ripening stages (MG, mature green; BR, breaker; MR, mature red) of E1 and E115 in the years 2013–2014. Ascorbic acid is expressed as mg /100 g FW. Total phenolics are expressed as mg GAE/100 g FW. Values are means ± SD. Values with different letters are significantly different (*p* < 0.05)

In order to in-depth understand the molecular mechanisms that regulate the biosynthesis and accumulation of antioxidants in tomato fruit, a whole transcriptome analysis of the two selected genotypes E1 and E115 has been undertaken. The two tomato genotypes were grown in open field and phenotypic and transcriptomic analyses of fruits were performed at three developmental stages: mature green (MG), breaker (BR) and mature red (MR). In Fig. 1 the average content of AsA and Phe at the three developmental stages recorded in the years 2013–2014 are reported. In E115 a higher content of ascorbic acid and total phenolics compounds was found at the three stages of ripening.

In order to better define the content of phenolics in the fruit of the two genotypes, an HPLC analysis was carried out (Table 1). This analysis demonstrated that both the content of phenolic acids and of flavonoids were generally higher in E115 fruits compared to E1 fruits. A significantly higher level of chlorogenic acid, 5-caffeolquinic acid, rutin and chalconaringenin was recorded in E115 compared to E1. In particular in E115 chalconaringenin level, which was very low in E1 in all the ripening stages, reached 8.43 ± 2.47 mg/100 g and 5.72 ± 0.43 mg/100 g at the breaker and mature red stage, respectively. The content of others phenolics compounds detected by HPLC analysis was not significantly different in the two genotypes.

**Table 1:** Phenolic compounds amount (mg/100 g FW) quantified by HPLC analyses

Phenolic compounds	E1			E115		
	MG	BR	MR	MG	BR	MR
Chlorogenic acid	12.01 ± 1.44	22.18 ± 3.58	10.28 ± 1.17	48.33 ± 3.81^***^	41.33 ± 3.43^***^	15.66 ± 3.17^*^
Ferulic acid	0.27 ± 0.02	0.34 ± 0.05	n.d.	0.15 ± 0.03^**^	n.d.	n.d.
5-Caffeoylquinic Acid	1.21 ± 0.10	0.84 ± 0.12	0.63 ± 0.23	1.77 ± 0.04	1.54 ± 0.15^**^	1.50 ± 1.01
Rutin	4.25 ± 0.30	2.74 ± 0.32	2.23 ± 0.46	7.58 ± 0.42^***^	5.50 ± 0.59^***^	4.63 ± 0.23^***^
Quercetin	0.17 ± 0.02	0.21 ± 0.06	n.d.	0.17 ± 0.02	0.19 ± 0.02	0.69 ± 0.08
Chalconaringenin	0.02 ± 0.01	0.06 ± 0.01	n.d.	0.18 ± 0.01^***^	8.43 ± 2.47^***^	5.72 ± 0.43
Kaempferol-3-rutinoside	n.d.	0.31 ± 0.03	0.18 ± 0.08	0.20 ± 0.01	0.93 ± 0.14^**^	0.82 ± 0.17^**^
Chalconaringenin-hexoside	n.d.	0.30 ± 0.02	0.22 ± 0.06	0.05 ± 0.01	0.31 ± 0.02	n.d.

Phenolic compounds were calculated in E1 and E115 fruits at three ripening stages (MG, mature green; BR, breaker; MR, mature red). Values are means ± SD. Asterisks indicate statistically significant differences of E115 compared to E1 (^*^p > 0.05, ^**^p < 0.01, ^***^p < 0.001)

### Transcriptome analysis of the genotypes E1 and E115

RNA sequencing experiment was performed on 18 RNA samples obtained from the genotypes E1 and E115. Sequencing was performed on RNA samples extracted from three biological replicates (named A, B, C) *per* genotype (E1 and E115) and *per* ripening stage (MG, BR, MR). Single-end RNA-seq strategy generated about 40 million of reads considering all the samples from the two genotypes at three developmental stages. After removing low quality reads and trimming adapter sequences, the high quality reads were retained for the different libraries.

The high quality reads were aligned against the *Solanum lycopersicum* reference genome using the software TopHat [33, 34]; only uniquely mapping reads were used for read counting (Additional file 3). After Reads processing, the quality of the experiment was evaluated on the basis of similarity between replicates by a PCA analyses and by the calculation of Euclidean distance between the samples and hierarchical clustering.

After reads count and HTS Filter analyses 19,332 (~ 56%) of the total tomato genes were retained for the differential expression analysis. As a result of these analyses we identified the differentially expressed genes (DEGs) between genotypes E115 and E1 at different ripening stages (Additional files 4, 5, and 6). At the mature green, breaker and mature red stages 3906, 2701, and 3611 differentially expressed genes were found, respectively (Additional file 7). Out of the 10,218 total DEGs, 5606 genes were differentially expressed between the two tomato genotypes in only one stage, whereas 306 where common to the three stages analyzed, as shown in the Venn diagram (Additional file 7). Among the DEGs, 351 were unknown while 433 resulted annotated as transcription factor (TF) when seeking in the Plant Transcription Factor Database (http://planttfdb.cbi.pku.edu.cn/; [41]). In particular, the TF-families more represented were the bHLH (40 DEGs) and the MYB/MYB-related (53 DEGs) families (Additional file 8). In addition, searching all DEGs against the reference canonical pathways in the KEGG database, we identified 11 DEGs belonging to the ascorbate biosynthetic pathways and 18 to the phenylpropanoids biosynthetic pathways, that were differentially expressed at least in two of the three ripening stages analyzed (Table 2).

**Table 2:** Differentially expressed genes (DEGs) between E115 and E1 identified through RNA-seq analysis. Genes belonging to the ascorbate and phenylpropanoids biosynthetic pathways that were differentially expressed in at least two ripening stages are reported, including their fold change, EC numbers and gene function in the KEGG database

Gene Identifier (Solyc ID)	Log2 fold change E115 vs E1			EC number	Gene Function
	MG	BR	MR		
Ascorbic Acid pathway					
*Solyc02g084210*	–	2.25	2.30	4.2.1.47	GDP-mannose-4,6-dehydratase
*Solyc02g030230*	–	1.91	1.20	4.1.1.35	UDP-glucose 4-epimerase
*Solyc04g082140*	−3.00	−2.53	−2.39	1.10.3.3	Laccase-22/L-ascorbate oxidase
*Solyc07g052230*	− 2.94	−1.90	− 2.39	1.10.3.3	Laccase-22/L-ascorbate oxidase
*Solyc05g054760*	–	1.94	2.77	1.8.5.1	Dehydroascorbate reductase
*Solyc03g083730*	−4.16	−3.50	−3.92	3.1.1.11	Pectin methylesterase
*Solyc09g091730*	–	4.87	7.10	3.1.1.11	Pectin methylesterase
*Solyc07g042390*	–	3.69	8.07	3.1.1.11	Pectin methylesterase
*Solyc07g064170*	–	2.44	3.20	3.1.1.11	Pectin methylesterase
*Solyc10g080210*	9.98	3.26	6.04	3.2.1.15	Polygalacturonase A
*Solyc06g071330*	−1.53	3.37	1.92	N.D.	Nuclease ascorbate transporter
Phenylpropanoid pathway					
*Solyc09g091510*	6.28	9.24	9.98	2.3.1.74	Chalcone synthase 1
*Solyc05g053550*	6.42	10.40	9.79	2.3.1.74	Chalcone synthase 2
*Solyc05g010320*	−3.38	1.86	1.85	5.5.1.6	Chalcone-flavonone isomerase
*Solyc05g052240*	3.90	7.33	6.19	5.5.1.6	Chalcone-flavonone isomerase
*Solyc02g083860*	2.71	6.46	7.31	1.14.11.9	Flavanone-3-hydroxylase
*Solyc02g089770*	2.65	6.61	4.12	1.1.1.195	Dihydroflavonol-4-reductase
*Solyc05g051020*	−2.60	6.20	5.13	1.1.1.219	Dihydroflavonol-4-reductase
*Solyc05g051010*	−2.70	2.97	4.41	1.1.1.219	Dihydroflavonol-4-reductase
*Solyc11g013110*	1.81	5.77	5.36	1.14.11.23	Anthocyanidin synthase
*Solyc03g078720*	–	3.82	2.03	2.4.1.215	Glucosyltransferase-2
*Solyc04g008330*	−5.57	4.01	2.94	2.4.1.215	Glucosyltransferase
*Solyc05g052870*	–	−2.63	−2.97	2.4.1.215	UDP-glucosyltransferase
*Solyc10g083440*	–	3.37	3.71	2.4.1.115	UDP flavonoid 3-O-glucosyltransferase
*Solyc11g007390*	5.19	−2.11	−1.81	2.4.1.177	UDP-glucosyltransferase
*Solyc10g050160*	−3.13	−2.13	−2.20	2.1.1.104	Caffeoyl-CoA 3-O-methyltransferase
*Solyc08g076790*	3.19	2.83	3.81	1.1.1.219	Cinnamoyl-CoA reductase
*Solyc08g076780*	6.04	10.53	4.21	1.2.1.44	Cinnamoyl-CoA reductase
*Solyc04g080550*	2.18	1.74	3.20	1.3.1.45	Phenylcoumaran benzylic ether reductase

**Fig. 2:**
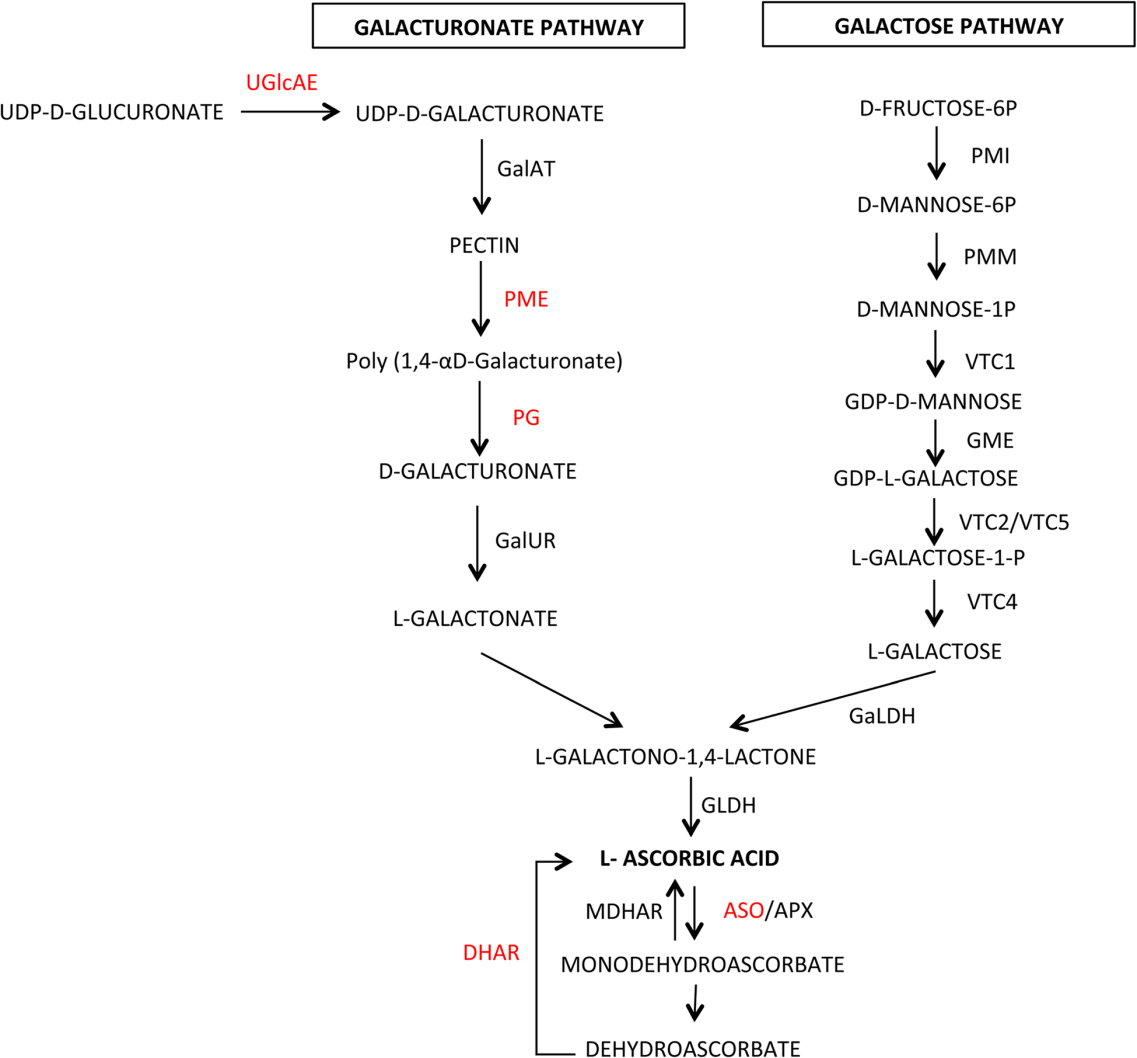
Schematic model illustrating the ascorbic acid biosynthetic pathway. The enzymes whose expression was different in the genotypes E1 and E115 are indicated by a red font. UGlcAE, UDP-glucuronic-acid-4-epimerase; GalAT, galacturonosyltransferase; PME, pectin methylesterase; PG, polygalacturonase; GalUR, D-galacturonate reductase; GLDH, L-galactonolactone dehydrogenase; MDHAR, monodehydroascorbate; DHAR, dehydroascorbate reductase; ASO, L-ascorbate oxidase; APX, L-ascorbate peroxidase; PMI, mannose-6-phosphate isomerase; PMM, phosphomannose mutase; VTC1, GDP-D-mannose pyrophosphorylase GME, GDP-D-mannose 3′,5′-epimerase; VTC2/VTC5 GDP-L-galactose-phosphorylase; VTC4, L-galactose-1-P phosphatase; GalDH, L-galactose dehydrogenase

Among the structural genes of the ascorbic acid pathways (Fig. 2), we identified one gene involved in the GDP-L-fucose biosynthesis (*Solyc02g084210*), coding for a GDP-mannose-4,6-dehydratase, which was up-regulated in E115 vs E1 in the BR and MR stages and that could be involved in alternatives AsA biosynthetic pathways. The other structural genes identified belong to the alternative galacturonate pathway or to the translocation and recycling pathways. Interestingly we identified two genes involved in the galactose pathway: the *GDP-D-mannose-3′5’-epimerase 1* and *2* (*Solyc01g097340* and *Solyc09g082990)* [42, 43]. However, the gene *Solyc01g097340* was up-regulated in E115 vs E1 only at the MR stage; and, the gene *Solyc09g08299* was down-regulated in E115 vs E1 only at the MG stage (Additional files 4 and 6). By investigating the metabolic pathway database SolCyc (https://solgenomics.net/tools/solcyc/; [44]) and by using information on the *S. lycopersicum* genes of the different ascorbate pathways recently identified [13] we confirmed the involvement of four *PME* isoforms (*Solyc03g083730*, *Solyc09g091730*, *Solyc07g042390* and *Solyc07g064170*) and one *polygalacturonase A* (*PG*; *Solyc10g080210*) in the galacturonate biosynthetic pathway. Two identified *laccase-22/L-ascorbate-oxidase homolog* (*LAC1*; *Solyc04g082140* and *Solyc07g052230*) and one *dehydroascorbate reductase* (*Solyc05g054760*) might enter the recycling AsA pathway, whereas one *nucleobase ascorbate transporter* (*Solyc06g071330*) might have a role in the transport of ascorbic acid in different intracellular compartments. Interestingly the two genes coding for LAC1 resulted down-regulated in E115 vs E1 in all the ripening stages. All the other genes, outside of one gene coding for the PME Solyc03g083730*,* resulted up-regulated in E115 in the last two stages of ripening.

**Fig. 3:**
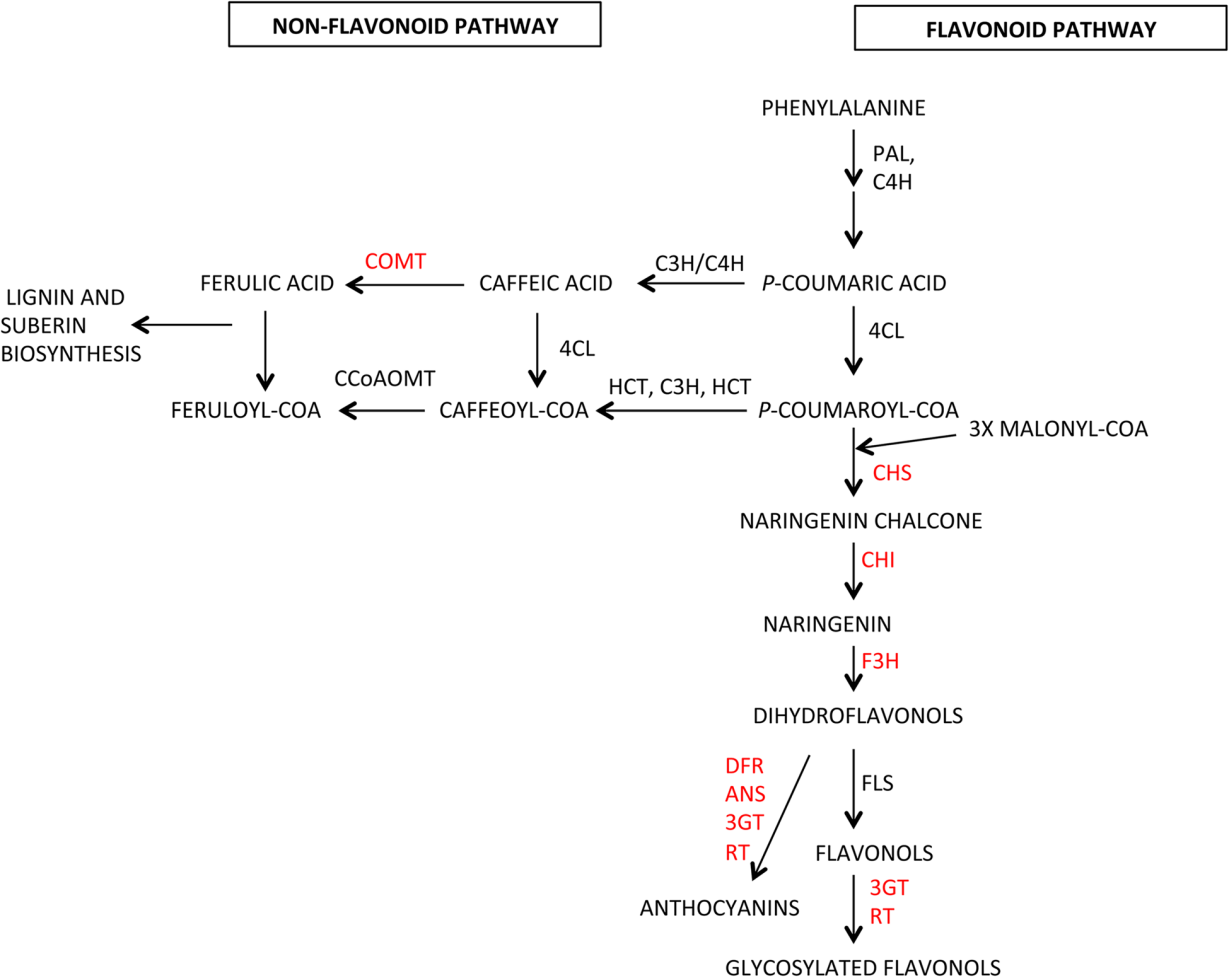
Schematic model illustrating the phenylpropanoids biosynthetic pathway. The enzymes whose expression was different in the genotypes E1 and E115 are indicated by a red font. PAL, Phenylalanine-ammonia-lyase; C4H, cinnamate 4-hydroxylase; 4CL, 4-coumarate: CoA ligase; HCT, cinnamoyl-CoA shikimate/quinate transferase; C3H, *p*-coumaroyl ester 3-hydroxylase; CHS, chalcone synthase; CHI, chalcone isomerase; F3H, flavanone-3-hydroxylase; FLS, flavonol synthase; 3GT, flavonoid-3-O-glucosyltransferase; RT, flavonoid 3-*O*-glucoside-rhamnosyltransferase; DFR, dihydroflavonol reductase; ANS, anthocyanidin synthase; COMT, caffeic/5-hydroxyferulic acid O-methyltransferase; CCoAOMT, caffeoyl-CoA O-methyltransferase

As for the phenylpropanoids pathway (Fig. 3), 14 out of the 18 identified genes belong to the flavonoids biosynthetic pathway including those encoding the chalcone synthases 1 and 2 (CHS), the chalcone isomerase, the flavanone-3-hydroxylase, the dihydroflavonol reductase, the anthocyanidin synthase, and the flavonoid glycosyltransferase. Of these 14 genes, 12 genes were up-regulated in E115 vs E1 at the breaker and mature red stages. In particular the genes coding for CHS1 and CHS2 (*Solyc05g053550* and *Solyc09g091510*) were strongly up-regulated in all the ripening stages. The four other DEGs belong to the lignin biosynthetic pathways (two *cinnamoyl-CoA reductase*, one *phenylcoumaran benzylic ether reductase* and one *caffeoyl-CoA-3-methyltransferase*) the gene *Solyc10g050160* coding for a caffeoyl-CoA-3-O-methyltransferase resulted down-regulated in E115 vs E1 in all the ripening stages.

### Identification of antioxidant-associated genes by co-expression network analysis

An alternative analysis tool, WGCNA (weighted gene co-expression  network analysis), was adopted for clarifying the molecular mechanisms that regulate the biosynthesis and accumulation of AsA and phenolics in tomato fruit and for finding new genes associated with antioxidants production. Co-expression networks were constructed on the basis of pairwise correlations between genes and their common expression trends across all samples. This analysis resulted in 67 distinct modules (each labeled with a colour) showed in the dendrogram in Additional file 9. The grey module was reserved for unassigned genes and does not represent a real module. The list of the genes assigned to each module and their measure of module membership (MM) is in Additional file 10. In total, 1840 genes were grouped in the grey module, while the turquoise and plum modules showed the maximum (2043) and minimum (37) number of genes, respectively.

**Fig. 4:**
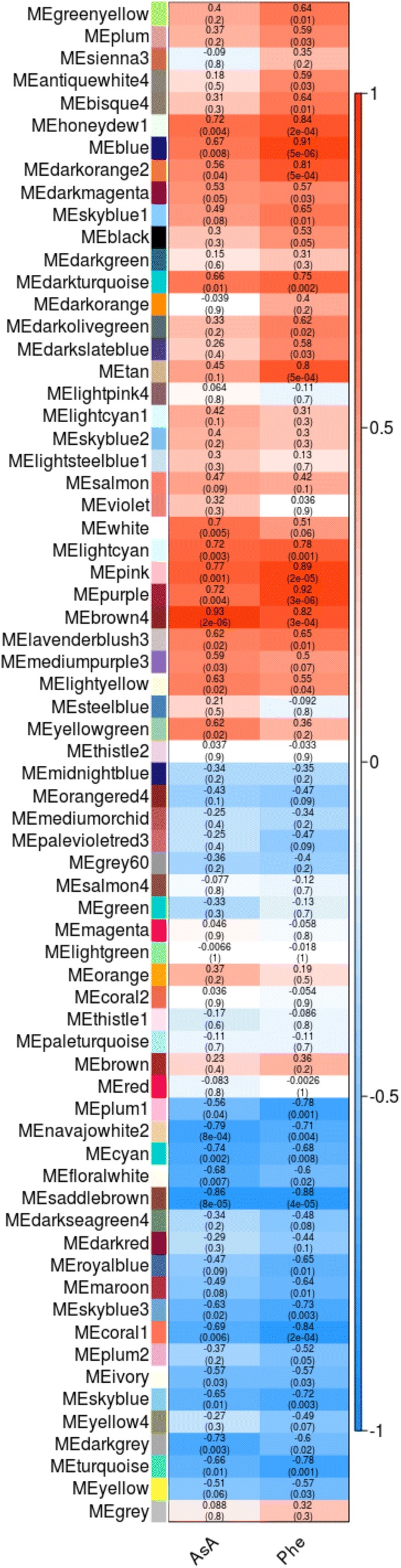
Weighted gene co-expression network analysis of antioxidants associated genes. In figure are reported the correlation between module and antioxidants compounds and corresponding *p*-values. The left panel shows the modules. The right panel is a color scale for module-trait correlation from − 1 to 1

Association of each co-expression module with each metabolite was quantified by Pearson’s correlation coefficient analysis and visualized in a heat map (Additional file 9 and Fig. 4). The analysis identified the several significant module-trait associations. Interestingly, we found 12 and 20 modules positively correlated with AsA and phenolics, respectively, while 11 and 17 were negatively correlated. Furthermore, most of the modules significantly correlated to AsA and Phe content were in common. Successively, we quantified associations of individual genes with the studied traits, by defining Gene Significance (GS). In particular, we concentrated on genes with a Gene Significance (GS) that is > 0.8 or < − 0.8 (i.e. a negative or a positive correlation greater than 0.8) and with a *p* < 0.001. The relationships between the modules and the metabolites were also investigated by generating a dendrogram and an adjacent heat-map (Additional files 11 and 12). These results are consistent with the correlation matrix described before. For AsA, the eigengene dendrogram and the heat-map indicated that four modules (brown4, lightcyan, pink and purple) were highly related among them and with the metabolite. Considering phenylpropanoids, three modules (pink, purple and blue) were strongly correlated.

Searching among genes present in each module correlated with the metabolite of interest, we found 41 DEGs coding for transcription factors strictly related to the content of ascorbic acid and phenylpropanoids (GS > ± 0.8; *p* < 0.001) (Table 3, Additional file 13). Among the identified TFs, the expression of genes codifying for three zinc finger proteins (*Solyc10g079120*, *Solyc10g080260* and *Solyc01g087170*), for a FAR1 (*Solyc01g112320*) and for a B3 (*Solyc01g108140*) was related with the ascorbic acid content in ripening fruits. As for the phenylpropanoids pathway, the analyses carried out identified TFs that have been reported to be involved in the regulation of flavonoid biosynthesis such as the TFs bHLH, NAC and MADS box [22, 45]. In the same submodules related to ascorbic acid and phenylpropanoids in which the TFs were found, the presence of antioxidants pathway related genes was also evidenced (Additional files 10 and 14). Indeed, in the blue module, that is strictly related to phenylpropanoids content, we could also detect the presence of several genes coding for enzymes of this metabolic pathway. Moreover, in the brown modules related to AsA content, one gene coding for an ascorbate peroxidase (*Solyc02g083620*) was evidenced. One hydroxycinnamoyl CoA shikimate/quinate hydroxycinnamoyltransferase (*Solyc07g005760*), one malonyl CoA anthocyanin 5-O-glucoside-6-O-malonyltransferase (*Solyc10g008650*) and one Polygalacturonase A (*Solyc10g080210*) were instead found in the pink modules related to both AsA and phenolics. Finally, in the AsA and phenolics-related purple modules a UDP-glucosyltransferase (*Solyc01g107850*), a polygalacturonase (*Solyc07g042160*), an ascorbate peroxidase (*Solyc09g007270*) and, also, the Laccase 22/L-ascorbate oxidase coded by the gene *Solyc07g052230*, were found. These findings further validate the potential role of the transcription factors identified by the WGCNA analyses.

**Table 3:** List of transcription factors related to ascorbate and phenolics accumulation identified through WGCNA analysis

Gene Identifier (Solyc ID)	Description	Module Color	GS.Phe	p.GS.Phe	GS.AsA	p.GS.AsA
*Solyc01g108140*	B3	brown4			−0.898	1.3E-05
*Solyc01g112320*	FAR1	brown4			0.893	1.7E-05
*Solyc10g080260*	Zinc finger	brown4			0.887	2.3E-05
*Solyc01g087170*	Zinc finger	brown4			0.885	2.7E-05
*Solyc06g076290*	GRAS	pink			0.823	0.0002
*Solyc10g079120*	Zinc finger	pink	0.828	3E-04	0.893	1.7E-05
*Solyc04g056320*	Zinc finger	pink	−0.869	5.4E-05		
*Solyc04g074700*	HD-ZIP	purple	−0.837	0.0002		
*Solyc08g061140*	HB-other (ISS)	purple	0.804	0.0005		
*Solyc07g052700*	MADS-box	purple	0.844	0.0001		
*Solyc12g100140*	bHLH	blue	−0.948	2E-07		
*Solyc12g096500*	CONSTANS	blue	−0.923	3E-06		
*Solyc07g052960*	GRAS	blue	0.9214	3E-06		
*Solyc12g008350*	ERF4	blue	−0.918	4E-06		
*Solyc10g006880*	NAC/NOR	blue	0.917	4E-06		
*Solyc02g063520*	HD-ZIP	blue	−0.908	7 E-06		
*Solyc06g069430*	FUL1	blue	0.907	8E-06		
*Solyc08g061130*	HY5	blue	0.906	8 E-06		
*Solyc10g079050*	bHLH	blue	0.852	0.0001		
*Solyc09g065660*	HSF A3	blue	0.901	1 E-05		
*Solyc03g121840*	Histone H1	blue	0.897	1 E-05		
*Solyc07g043580*	bHLH	blue	−0.897	1 E-05		
*Solyc10g084180*	C2H2L	blue	−0.892	2–05		
*Solyc06g049040*	BZIP	blue	−0.885	3E-05		
*Solyc08g077230*	APRR2	blue	0.877	4–05		
*Solyc02g092550*	LOB	blue	−0.876	4 E-05		
*Solyc02g077840*	ERF12	blue	−0.871	5–05		
*Solyc06g036170*	SCL	blue	0.868	6E-05		
*Solyc06g053960*	HSF A3	blue	0.866	6 E-05		
*Solyc01g104650*	BZIP	blue	0.845	0.0001		
*Solyc02g090820*	HSF	blue	0.847	0.0001		
*Solyc11g017470*	NAC4	blue	0.837	0.0002		
*Solyc04g080740*	BZIP	blue	0.825	0.0003		
*Solyc09g007990*	TERF 5	blue	0.823	0.0003		
*Solyc01g102260*	PHE1	blue	−0.818	0.0004		
*Solyc06g061030*	ARR11	blue	−0.816	0.0003		
*Solyc06g074120*	BLH1	blue	0.811	0.0004		
*Solyc08g008380*	ARF 9	blue	−0.810	0.0004		
*Solyc03g044300*	AP2A	blue	0.807	0.0005		
*Solyc01g079870*	NFYC-2	blue	0.804	0.0005		
*Solyc12g007300*	B3	blue	0.801	0.0006		

In the Table are shown values of GS (Gene Significance) > ± 0.8

### Analyses of the expression of selected candidate genes in cultivated tomato genotypes by RT-qPCR

**Fig. 5:**
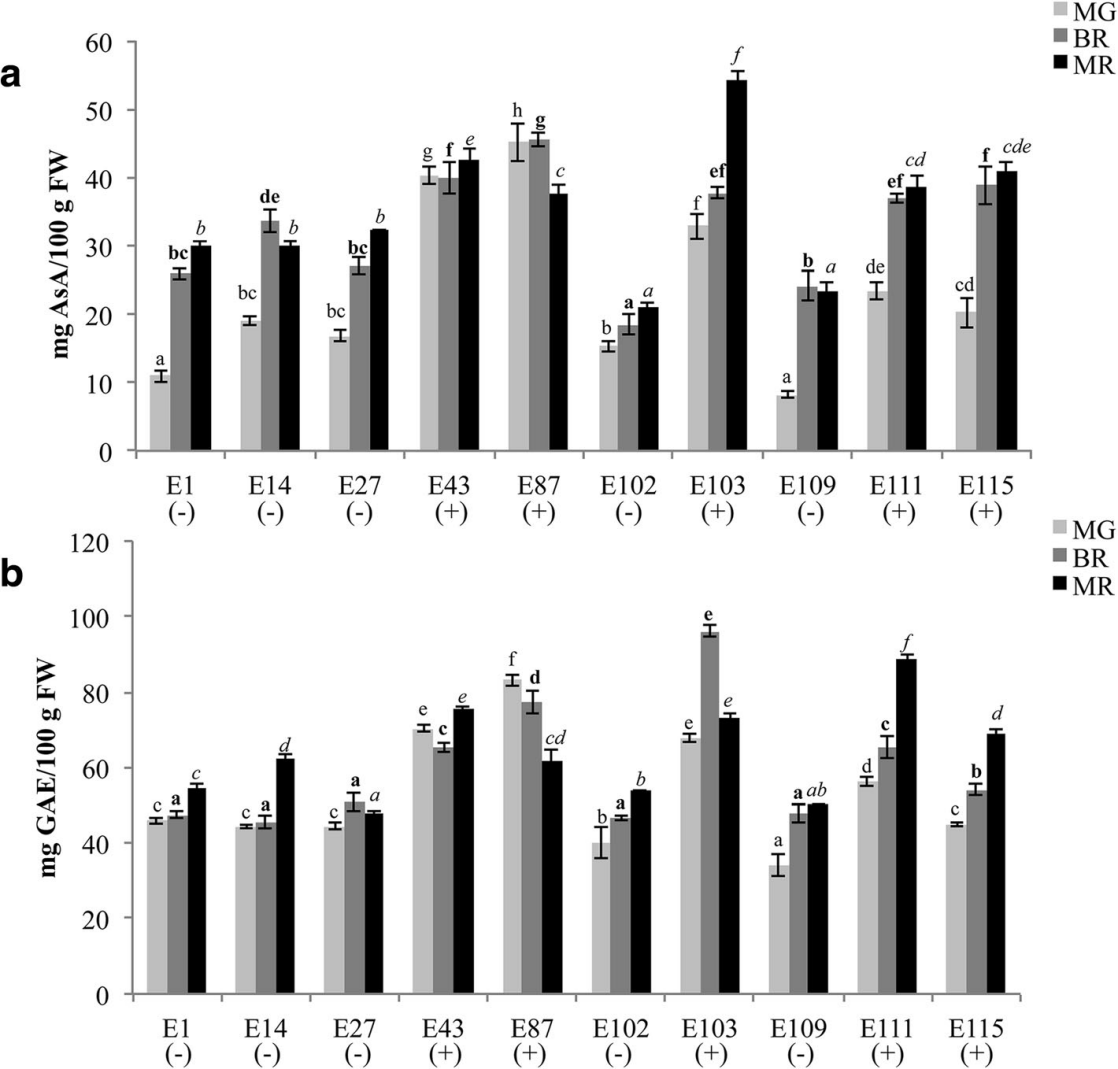
Content of ascorbic acid (**a**) and total phenolics (**b**) in tomato fruits. Antioxidant levels were calculated in fruits at different ripening stage (MG, mature green; BR, breaker; MR, mature red) of E1, E14, E27, E43, E87, E102, E103, E109, E111 and E115 in the years 2015–2016. Ascorbic acid is expressed as mg /100 g FW. Total phenolics are expressed as mg GAE/100 g FW. Values are means ± SD. Values with different letters in each ripening stage are significantly different (*p* < 0.05). Normal, bold and italics letter styles indicate significance in MG, BR and MR stages, respectively

The content of total phenolics and ascorbic acid was determined in ten cultivated tomato genotypes, including E1 and E115, classified as low-metabolites content and high-metabolites content, respectively, in previous evaluations carried out in red ripe fruits [40]. The analyses here carried out on fruits at different ripening stages (MG, BR, MR) in the years 2015–2016 further confirmed that the genotypes E1, E14, E27, E102 and E109 were characterized by a low content of both ascorbic acid and phenylpropanoids in tomato fruits. On the contrary, the genotypes E43, E87, E103, E111 and E115 contained a high content of bioactive compounds in ripening tomato fruits (Fig. 5a, b).

We further carried out an expression analysis by RT-qPCR of selected structural genes and TFs identified by RNA-seq and WGCNA analyses comparing E1 and E115. The candidate genes (CGs) whose expression was analyzed by RT-qPCR are reported in Additional file 2. The list includes the genes *dehydroascorbate reductase (Solyc05g054760)*, *GDP-mannose-4,6-dehydratase (Solyc02g084210)*, *Nuclease ascorbate transporter (Solyc06g071330)*, *PME (Solyc09g091730)* and *PG (Solyc10g080210)*. These genes did not evidence significant differences between E1 and E115 (data not shown).

**Fig. 6:**
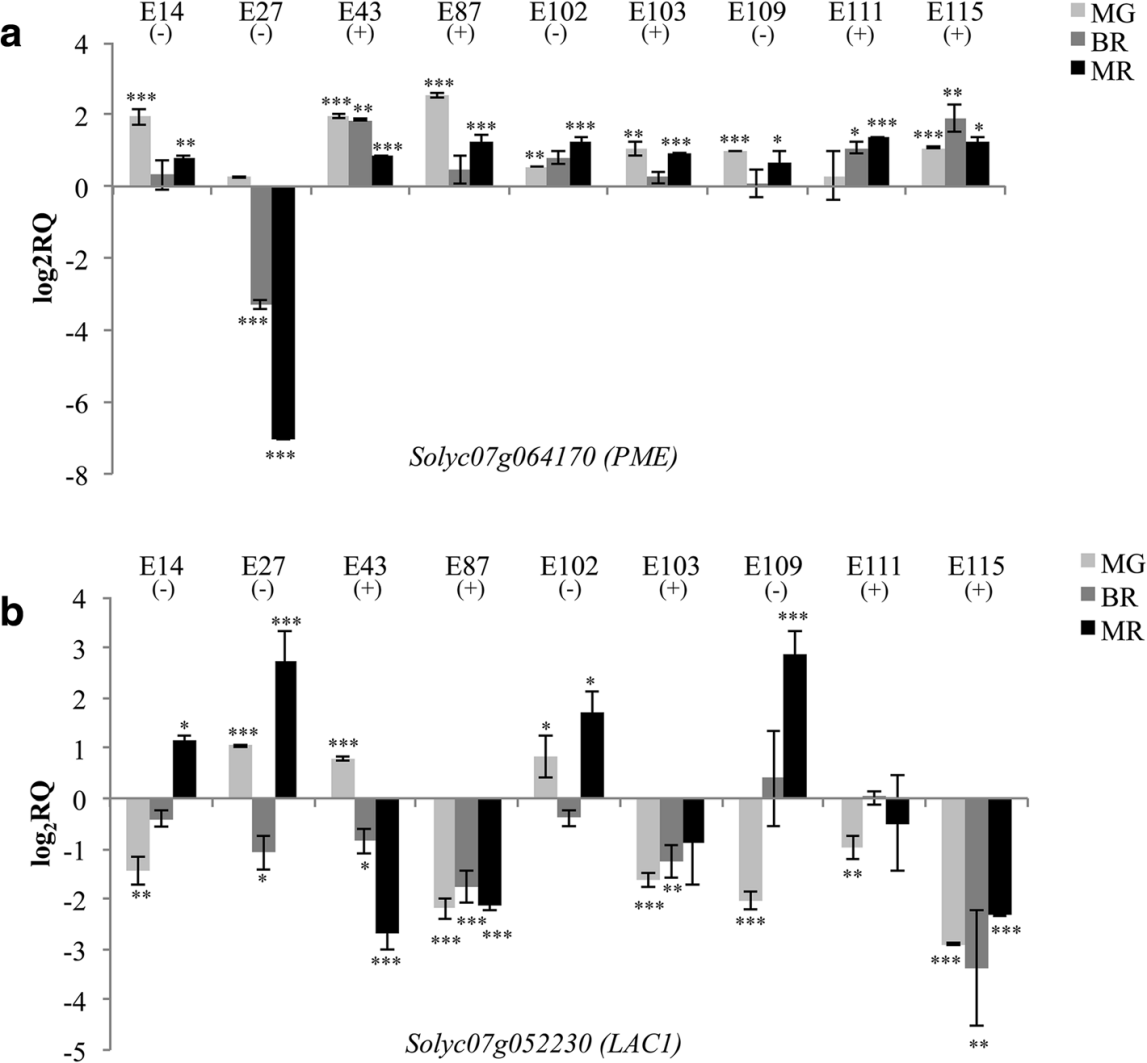
RT-qPCR expression analyses of the identified candidate genes controlling AsA accumulation in tomato fruits. The expression levels of **a** *Solyc07g064170* (*PME*); and **b** *Solyc07g052230* (*LAC1*) are reported in comparison to that observed in E1. Asterisks indicate statistically significant differences of each genotype compared to E1 (*p < 0.05, **p < 0.01, ***p < 0.001)

**Fig. 7:**
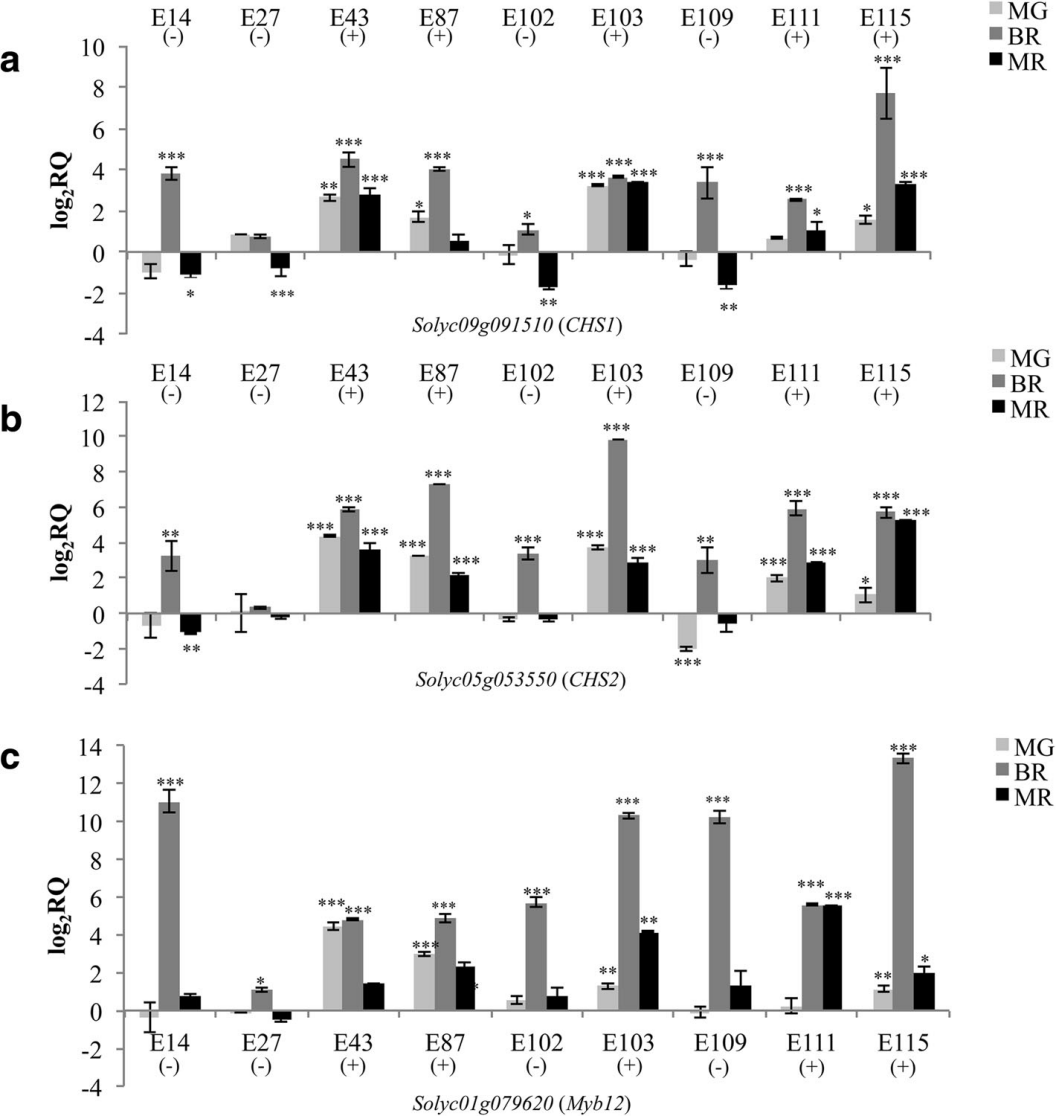
RT-qPCR expression analyses of the identified candidate genes controlling phenolics accumulation in tomato fruits. The expression levels of **a** *Solyc09g091510* (*CHS1*), **b** *Solyc05g053550* (*CHS2*) and **c** *Solyc01g079620* (*Myb12*) are reported in comparison to that observed in E1. Asterisks indicate statistically significant differences of each genotype compared to E1 (*p < 0.05, **p < 0.01, ***p < 0.001)

**Fig. 8:**
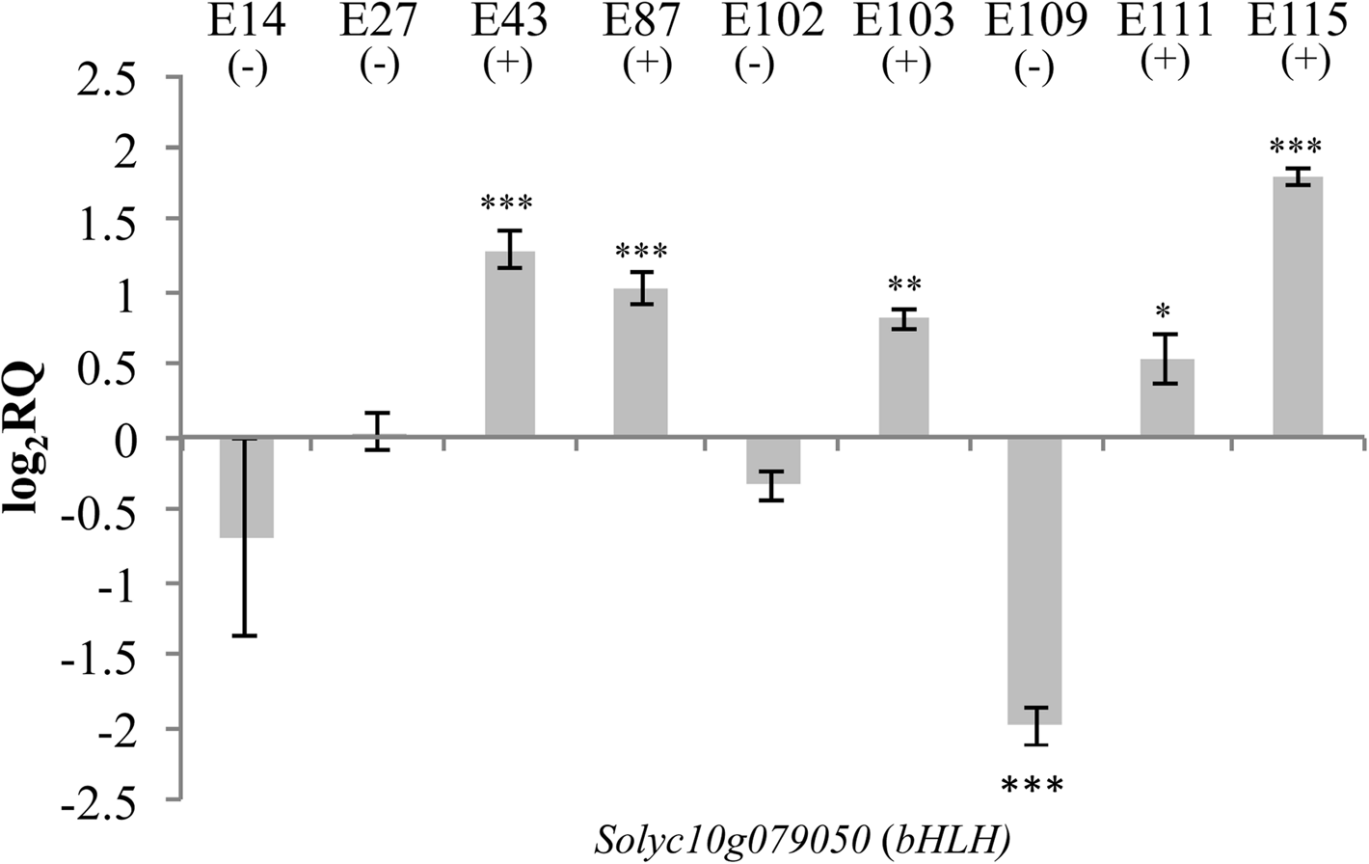
RT-qPCR expression analyses of the gene *Solyc10g079050* (*bHLH)* in mature green tomato fruits. The expression levels are reported in comparison to that observed in E1. Asterisks indicate statistically significant differences of each genotype compared to E1 (*p < 0.05, **p < 0.01, ***p < 0.001)

The expression of the CGs that in the RT-qPCR analyses showed a significant difference in E115 vs E1 was further verified also in the other cultivated tomato genotypes classified as low-metabolites content (E14, E27, E102, E109) and high-metabolites content (E43, E87, E103, E111), respectively (Figs. 6, 7, 8).

The RT-qPCR analysis demonstrated that *PME Solyc07g064170* was up-regulated in E115 compared to E1 in all the ripening stages. Interestingly, this gene resulted up-regulated in at least two stages of ripening in all the genotypes compared to E1, whereas resulted strongly down-regulated in the BR and MR stages in one genotype (E27) with a low level of AsA in the fruit (Fig. 6a).

We also analyzed the expression of the *Laccase 22*/*L-ascorbate oxidase* coded by the gene *Solyc07g05230*, which might enter the recycling pathway of AsA by reducing L-ascorbate into monodehydroascorbate. In all the genotypes here analyzed the gene *Solyc07g05230* resulted up-regulated in the mature red stage of all the low-AsA cultivars, while it was down-regulated in all the high-AsA cultivars. In the high-AsA genotypes E87, E103 and E115 the gene *Solyc07g05230* resulted down-regulated in all ripening stages (Fig. 6b).

As for the phenylpropanoids, HPLC analyses demonstrated that there was a higher content of naringenin chalcone in E115 vs E1 at the breaker and mature red stages. Accordingly, RNA-seq analyses demonstrated an up-regulation of the genes *Solyc09g091510* and *Solyc05g053550* coding for the enzymes CHS1 and CHS2, respectively, controlling naringenin chalcone synthesis. In all the genotypes, the expression of both *CHS1* and *CHS2* peaked at the breaker stages and then decreased again in mature red fruit in accordance with previous results [6]. The RT-qPCR analyses demonstrated that both the genes coding for CHS were up-regulated in all the ripening stages in the high-phenolics cultivars compared to E1, while they were up-regulated only in the breaker stage in the low-level cultivars (Fig. 7a, b). Next, we analyzed the expression of the TF Myb12, which was up-regulated in E115 vs E1 following the RNA-seq analyses and is known to directly control *CHS* expression [10]. Consistent with results obtained for the *CHS* genes, the expression of *Myb12* picked at the breaker stage in all the genotypes analyzed (Fig. 5c). Moreover, the expression of *Myb12* was higher in the high-phenolics cultivars E43, E87, E103 and E115 compared to E1 in all the ripening stages, and in E111 vs E1 in the last two stages of ripening. In the low-phenolics cultivars compared to E1 a higher expression of *Myb12* was evidenced only in the breaker stage.

Among the TFs identified by WGCNA analyses, we investigated through RT-qPCR the expression of one *bHLH* gene (*Solyc10g079050*) in all the cultivated genotypes here tested (Fig. 8). In mature green fruits, the TF *bHLH* resulted up-regulated in E115 and in all the other high-antioxidants cultivars, whereas all the low-antioxidants genotypes exhibited expression levels comparable to E1. The *bHLH* gene resulted down-regulated in the low-antioxidants cultivars E109 compared to E1. In the other ripening stages the expression of the *bHLH* gene did not correlate with the accumulation of the metabolites in the fruit of the different genotypes (data not shown).

## Discussion

Nowadays, the majority of the enzymes of the biosynthetic pathways and of the structural genes controlling the production and the accumulation of antioxidants in plants are known; however, the mechanisms that regulate the expression of these genes are yet to be investigated. In some cases, changes in the expression of only one transcription factors and/or regulator gene of the antioxidants biosynthetic pathways could modify the content of the compounds accumulating in the fruit or in other plant organs [6]. For these reasons, herein we investigated in diverse genotypes the different expression level of genes in tomato fruits that may regulate the content of ascorbic acid and phenolics. Indeed, the presence of one allelic variant in the promoter of one structural and/or regulator gene could explain specific or common mechanisms controlling the accumulation of these metabolites in the fruit. RNA-seq based transcriptome analysis has been shown to be an efficient tool in order to identify genes involved in antioxidant biosynthesis in several plants such as plum, strawberry and watermelon [22, 46]. For the same purpose, in the present study two contrasting cultivated genotypes (E1 and E115) were analyzed by RNA-seq to characterize the changes in gene expression in different stages of ripening. Furthermore, transcription factors that may control antioxidants accumulation were identified performing a weighted gene co-expression network analysis. For a group of selected candidate genes results obtained by RNA-seq analyses were further confirmed by RT-qPCR analyses carried out on a number of tomato genotypes that accumulate different amount of bioactive compounds in the fruit. Altogether, these analyses allowed identifying several structural and regulator genes of the ascorbic acid and of the phenylpropanoids biosynthetic pathways.

It was here demonstrated that a higher content of ascorbic acid accumulated in E115 compared to E1 in all the ripening stages. Transcriptome analyses demonstrated that in the cultivar E115 the higher accumulation of ascorbic acid in ripening tomato fruit was due to a higher expression of genes of the recycling and the galacturonate pathways and not of the main galactose pathway. Indeed, only one gene of the main galactose pathway (The GDP-D-mannose-3′5’-epimerase 1 *Solyc01g097340)* was found up-regulated in E115 vs E1 at the mature red stage. This is in line with previous studies conducted in high- and low- vitamin C tomato cultivars which demonstrated that, during ripening, differences in AsA accumulation were due mostly to differences in the rate of AsA turnover and recycling rather than to differences in the rate of AsA biosynthesis [47]. Furthermore, it has been postulated that in the last stages of tomato fruit ripening, the galacturonate pathway may contribute to the final AsA content [13, 18]. Indeed, the action of pectin methylesterases, that are responsible for the demethylation of pectin, may affect the release of pectin-derived D-galacturonic acid (GalUA) used as metabolic precursor of AsA biosynthesis [13, 18]. Interestingly, here we identified three *PMEs* and one *PG* that were up-regulated in E115 vs E1 in the last two stages of ripening. Among these genes, one *pectin methylesterase* (*PME* - *Solyc07g064170*) was identified that has been previously listed as involved in the D-Galacturonate pathway [13]. RT-qPCR analyses evidenced that in the genotype E115 this *PME* resulted up-regulated in all the ripening stages compared to the low-AsA genotype E1. Interestingly, the gene *Solyc07g064170* resulted up-regulated in all the cultivated genotypes here analyzed compared to E1 in at least two stages of ripening. One exception was the genotype E27, characterized by a low content of ascorbic acid in the fruit, in which the gene *Solyc07g064170* was strongly down-regulated in the last two stages of ripening. Therefore, it can be hypothesized that the down-regulation of the identified PME coded by *Solyc07g064170* may be involved in the low levels of AsA accumulation in the genotype E27.

Through RNA-seq analyses and WGCNA two *laccase-22/L-ascorbate oxidase* coded by the genes *Solyc07g052230* and *Solyc05g054760* were also identified that were down-regulated in the genotype E115 vs E1. Ascorbate oxidase is an enzyme that oxidases ascorbate to the unstable radical monodehydroascorbate (MDHA), which rapidly disproportionates to yield dehydroascorbate (DHA) and ascorbate contributing to the modulation of the AsA redox state [48]. In tomato, suppression of ascorbate oxidase expression promoted ascorbic acid accumulation in the fruit [48]. Furthermore, it has been demonstrated that a lower activity of an ascorbate oxidase could increase the concentration of the reduced form of ascorbate [49, 50]. Interestingly, the involvement of the enzyme LAC1 coded by *Solyc07g052230* in controlling AsA content in tomato fruits was previously hypothesized in one *Solanum pennellii* introgression line [16]. The role of the *LAC1 Solyc07g052230* in controlling AsA content in tomato fruits was further supported by RT-qPCR analyses here carried out which demonstrated that this gene was down-regulated in the fruit of all the genotypes that exhibited a high level of AsA and up-regulated in the fruit of all the low AsA genotypes. Main differences in the expression levels of the gene *Solyc07g052230* were evidenced in the tomato cultivars at the red ripe stages; this is in accordance with previous results that demonstrated that the recycling pathway contribute to final AsA content mostly in the last stage of ripening [47]. Recently, a diminution in ascorbate oxidase activity has been also demonstrated to affect carbon allocation and to improve yield in tomato under water deficit [51]. Therefore, in the future, it will be interesting to study the potential correlation between the down-regulation of *LAC1* in the cultivated tomato genotypes here analyzed and their improved response to oxidative stress.

As for phenylpropanoids accumulation, RNA-seq analyses demonstrated that in the genotype E115 there was a higher expression of the genes *Solyc09g091510* coding for CHS1 and of the gene *Solyc05g053550* coding for CHS2 compared to E1. Accordingly, in E115 vs E1 we detected a higher content of naringenin chalcone, a compound that accumulate in tomato fruit cuticle as a result of the ripening dependent increase in *CHS* expression [6]. Moreover, in E115 vs E1 there was a higher expression of other genes of the phenylpropanoids biosynthetic pathway: two *chalcone isomerase* (*CHI*), one *flavonone 3-hydroxylase* (*F3H*) and three *flavonoid glycosyltransferase* (*UGT*). In accordance, in E115 vs E1 the gene coding for Myb12 (*Solyc01g079620*), a TF known to activate the expression of the genes *CHS1*, *CHS2, CHI*, *F3H*, and *glycosyltransferases*, was also up-regulated [6, 52]. Therefore, the hypothesis is that in E115 the higher accumulation of phenolics compounds detected was mostly due to a higher expression of the TF *Myb12* modulating the expression of early flavonoid biosynthetic genes in the fruit. The pivotal role of Myb12 in controlling phenolics accumulation in tomato fruit was further confirmed by RT-qPCR that demonstrated that the expression of *Myb12* well correlated with the expression of the genes *CHS1* and *CHS2* in the high- and low-phenols cultivars. Therefore, altogether, it can be hypothesized that the level of phenolics compounds in tomato cultivars is mostly due to the transcriptional regulation of genes involved in the biosynthesis of these secondary metabolites.

In this paper, structural genes and transcription factors that are potentially associated with the biosynthesis of ascorbic acid and phenylpropanoids during fruit ripening were identified through a weighted gene co-expression network analysis (WGCNA). This analysis lead to the identification of weighted co-expression gene network modules significantly correlated with ascorbic acid and total phenolics. Among the genes present in each weighted module that correlated with the metabolite of interest, we found 41 DEGs coding for transcription factors strictly related to the content of ascorbic acid and phenylpropanoids.

Interestingly, the TF CCCH-type Zinc Finger *Solyc10g079120* was identified as potential regulator of both ascorbic acid and phenylpropanoids accumulation and may therefore be involved in the control of the accumulation of both these antioxidants in the fruit. The CCCH zinc finger proteins are generally known to play important roles in the regulation of plant growth, in developmental processes, and in the response to biotic and abiotic stresses [53, 54]. However, recently, when the transcriptomes of fruits at different developmental stages from two tomato cultivars were evaluated, a co-expression analysis revealed several CCCH-type zinc finger proteins whose expression patterns correlated with those of structural genes associated with ascorbic acid and flavonoids biosynthesis [20].

Out of the TFs positively related with ascorbate accumulation, one gene coding for a protein FAR1 (*Solyc01g112320*) was identified. Noteworthy, it was recently demonstrated that in *Arabidopsis thaliana* FAR1 regulates light-induced myo-inositol biosynthesis by transcription activation of myo-inositol-1-phosphate synthase [55] and may therefore be involved in an alternative ascorbate biosynthetic pathway.

Thirty-six transcription factors were identified in the WGCNA analyses related with phenolics accumulation, including TFs, such as bHLH, Zinc Finger and HY5, that are known to be important regulators of phenylpropanoids biosynthesis [20, 22, 44, 56]. For example, the TF long HYPOCOTYL5 (HY5) was previously found to regulate anthocyanin biosynthetic genes in *Arabidopsis thaliana* [57]. Moreover, the ripening-related TFs FUL1 (Solyc06g069430), NAC/NOR (Solyc10g006880) and APRR2 (Solyc08g077230), here identified, were previously found to be involved in the regulation of flavonoids accumulation [20, 57–59]. Indeed, transgenic tomato lines with reduced FUL or NAC/NOR expression had reduced expression level of the enzyme CHS [60, 61]. Other ripening-related TFs, such as the TFs NAC4 (Solyc11017470), and AP2a (Solyc03g044300) were evidenced among the transcription factors that may regulate phenolics accumulation [62–65]. The TFs GRAS (Solyc07g052960), bHLH (Solyc10g079050), AP2a (Solyc03g044300), NAC/NOR (Solyc10g006880), HB40-homolog (Solyc02g085630) and FUL1 (Solyc06g069430), found in the WGCNA analyses, were all recently recognized has direct targets of the tomato TF RIN, one of the earliest acting ripening regulators [60, 66]. Interestingly, it has been demonstrated that RIN positively regulates the expression of chalcone synthase, phenylalanine ammonia lyase1 and cinnamate-4-hydroxylase that are involved in the general phenylpropanoids and in the flavonoids pathways [67]. Therefore, the WGCNA analyses here carried out indicate that there is a strong relationship between phenylpropanoids biosynthesis and tomato fruit ripening, as previously hypothesized, even if the mechanism of this connection is still unclear [20, 61]. The idea that ripening-associated transcription factors may regulate also the synthesis of important strengthen by the RT-qPCR analyses here carried out. Indeed, our study demonstrated that the ripening-related bHLH TF coded by the gene *Solyc10g079050* resulted up-regulated in all the high-phenols cultivars and down-regulated in the low-phenols cultivar E109 at the mature green stage. Further genetic experimentation will now be required to validate the candidate regulators of phenylpropanoids accumulation in tomato fruit.

## Conclusion

Transcriptome analyses of tomato genotypes contrasting for antioxidants content in the fruit provided important information regarding candidate genes encoding biosynthetic enzymes and transcription factors involved in antioxidants biosynthesis. These analyses allowed us to identify one PME coded by the gene *Solyc07g064170*, which may affect the release of pectin-derived D-Galacturonic acid as metabolic precursor of ascorbate biosynthesis. In addition one gene, the *L-ascorbate oxidase Solyc07g052230*, which may favour the accumulation of reduced ascorbate in tomato fruits by controlling AsA redox state in the apoplast was identified. The pivotal role of the enzymes chalcone synthases coded by the genes *Solyc09g091510* and *Solyc05g053550* in controlling the accumulation of phenolic compounds in cultivated tomato genotypes and the transcriptional control of the *CHS* genes exerted by Myb12, was confirmed.

Finally, candidate regulators of the antioxidants accumulation in the fruit, such as several ripening-associated transcription factors, were also identified by a weighted gene co-expression network analysis. In particular, one bHLH TF coded by the gene *Solyc10g079050* might control antioxidants accumulation in mature green fruits of the low-antioxidants genotype E109. Further studies are now needed to better understand the role of the identified structural genes and transcription factors related to the biosynthesis of ascorbic acid and phenylpropanoids in tomato fruit.

In the future, specific breeding programs will be designed in order to combine multiple favorable genes here identified, such as *PME* and *LAC1* and/or *CHS* and *Myb12*, to stably increase the content of ascorbic acid and phenolics in novel tomato genotypes. The structural genes and regulators here identified could also be used as efficient genetic markers for selecting high antioxidant tomato cultivars.

## Additional files

Additional file 1: Description of the ten genotypes analyzed. The genetic background and geographical origin is reported together with traits related to fruit morphology, size and color. (XLSX 45 kb)

Additional file 2: Table showing the sequences of adopted primers for RT-qPCR. The sequences of adopted primers for amplifying the selected candidate genes and the housekeeping gene *EF-1α* are reported. (XLSX 10 kb)

Additional file 3: Table showing the percentage of uniquely mapped reads for each sample. (XLSX 48 kb)

Additional file 4: Table showing the list of differentially expressed genes at the Mature Green Stage. In a) List of the up-regulated genes between E115 and E1 fruit at the Mature Green stage and in b) List of the down-regulated genes between E115 and E1 fruit at the Mature Green stage. (XLSX 441 kb)

Additional file 5: Table showing the list of differentially expressed genes at the Breaker stage. In a) List of the up-regulated genes between E115 and E1 fruit at the Breaker stage and in b) List of the down-regulated genes between E115 and E1 fruit at the Breaker stage. (XLSX 311 kb)

Additional file 6: Table showing the list of differentially expressed genes at the Mature Red stage. In a) List of the up-regulated genes between E115 and E1 fruit at the Mature Red stage and in b) List of the down-regulated genes between E115 and E1 fruit at the Mature Red stage. (XLSX 412 kb)

Additional file 7: Analyses of differential expressed genes (DEGs) in E115 vs E1 in different stages of ripening. a) Numbers of DEGs up-regulated and down-regulated in E115 vs E1 in three stages of ripening (MG, Mature Green; BR, Breaker; MR, Mature Red) b) Venn diagram showing the numbers of non-overlapped and overlapped DEGs comparing three stages of ripening (MG, Mature Green; BR, Breaker; MR, Mature Red). (TIF 860 kb)

Additional file 8: Table showing the list of the transcription factors differentially expressed in E115 compared to E1. (XLSX 65 kb)

Additional file 9: Weighted gene co-expression network analysis of antioxidants-associated genes. Hierarchical cluster tree showing the modules of co-expressed genes. Each associated gene is represented by a leaf in the tree, while each module by a major tree branch. The lower panel in the figure shows the modules with assigned colours. (TIF 1403 kb)

Additional file 10: Table showing the list of *S. lycopersicum* genes assigned to the modules. The table shows the modules identified by WGNA analysis and their measure of module membership (MM). (ZIP 12845 kb)

Additional file 11: Visualization of the eigengene network representing the relationships among modules and Ascorbic Acid (AsA) trait. Upper panel: hierarchical clustering dendrogram of the eigengenes. Lower panel: heatmap showing eigengenes adjacency. (TIF 2370 kb)

Additional file 12: Visualization of the eigengene network representing the relationships among modules and Phenolics (Phe) trait. Upper panel: hierarchical clustering dendrogram of the eigengenes. Lower panel: heatmap showing eigengenes adjacency. (TIF 2398 kb)

Additional file 13: Table showing the average rpkm values of the transcription factors identified by Weighted gene co-expression network analysis. (XLSX 59 kb)

Additional file 14: Table showing in each module identified by WGNA analysis the list of transcription factors and antioxidants-related genes. (DOCX 74 kb)

## Abbreviations

4CL: 4-coumaroyl-CoA-ligase
AsA: Ascorbic acid
bHLH: Basic helix-loop-helix
BR: Breaker
C4H: Cinnamate-4-hydroxylase
CHS: Chalcone synthase
CNCD: Chronic non-communicable disease
CVD: Cardiovascular disease
DEG: Differentially expressed gene
DHA: Dehydroascorbate
F3H: Flavonone 3-hydroxylase
FW: Fresh weight
GalUA: D-Galacturonic acid
GS: Gene significance
LAC1: Laccase-22/L-ascorbate-oxidase homolog
MDHA: Monodehydroascorbate
MG: Mature green
MR: Mature Red
PAL: Phenylalanine ammonia lyase
PG: Polygalacturonase
Phe: Total phenolics
PME: Pectin methylesterase
RNA-seq: RNA sequencing
TF: Transcription factor
UGT: Flavonoid glycosyltransferase
WGCNA: Weighted gene co-expression network
