# Supplementary figures and images for: New insights in the control of antioxidants accumulation in tomato by transcriptomic analyses of genotypes exhibiting contrasting levels of fruit metabolites
Source: BMC Genomics. 2019 Jan 15;20:43. doi: 10.1186/s12864-019-5428-4 (PMC6332538; doi:10.1186/s12864-019-5428-4)

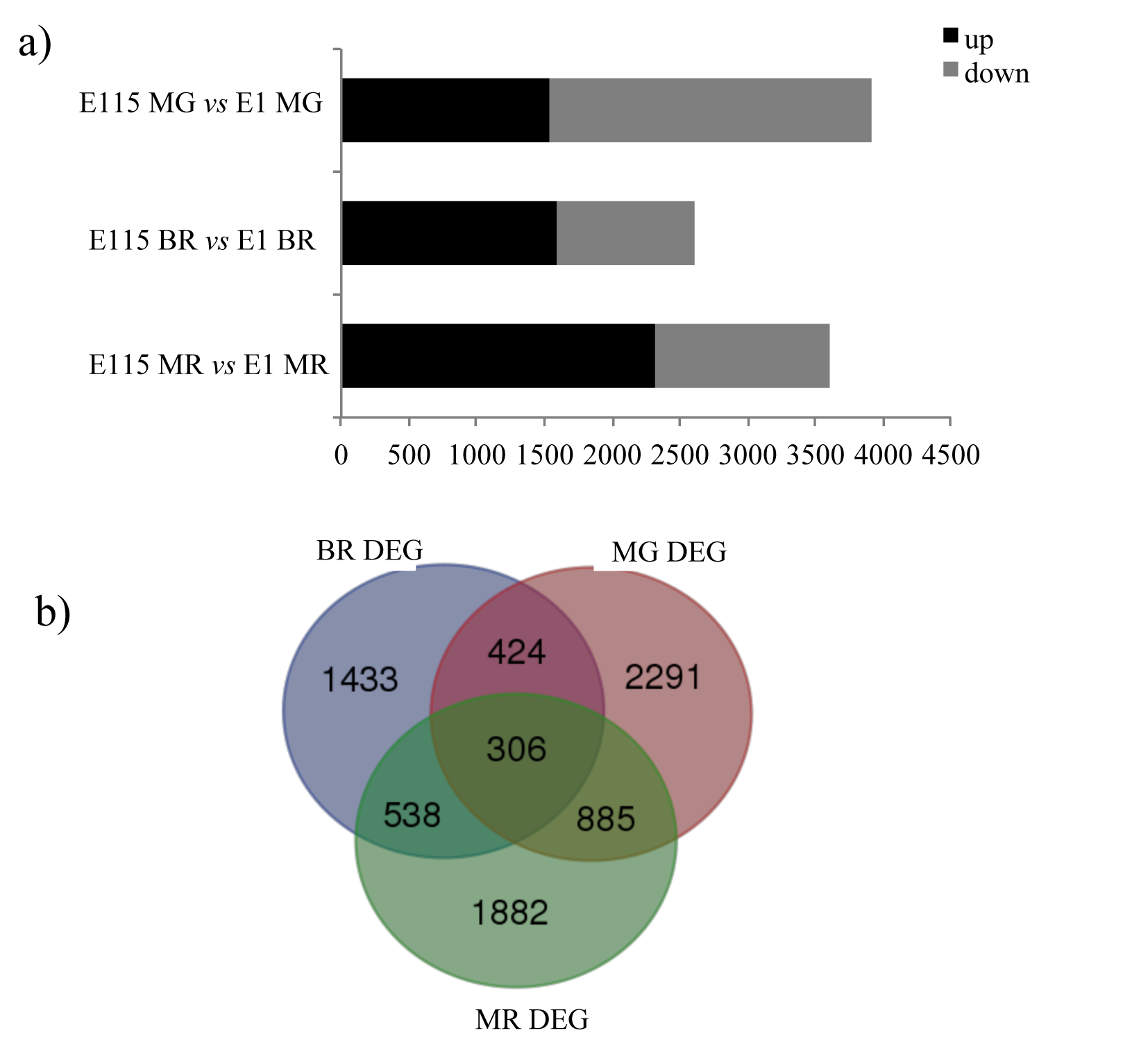

Supplement: Supplementary file 7 — Analyses of differential expressed genes (DEGs) in E115 vs E1 in different stages of ripening. a) Numbers of DEGs up-regulated and down-regulated in E115 vs E1 in three stages of ripening (MG, Mature Green; BR, Breaker; MR, Mature Red) b) Venn diagram showing the numbers of non-overlapped and overlapped DEGs comparing three stages of ripening (MG, Mature Green; BR, Breaker; MR, Mature Red). (TIF 860 kb) [file 12864_2019_5428_MOESM7_ESM.tif]

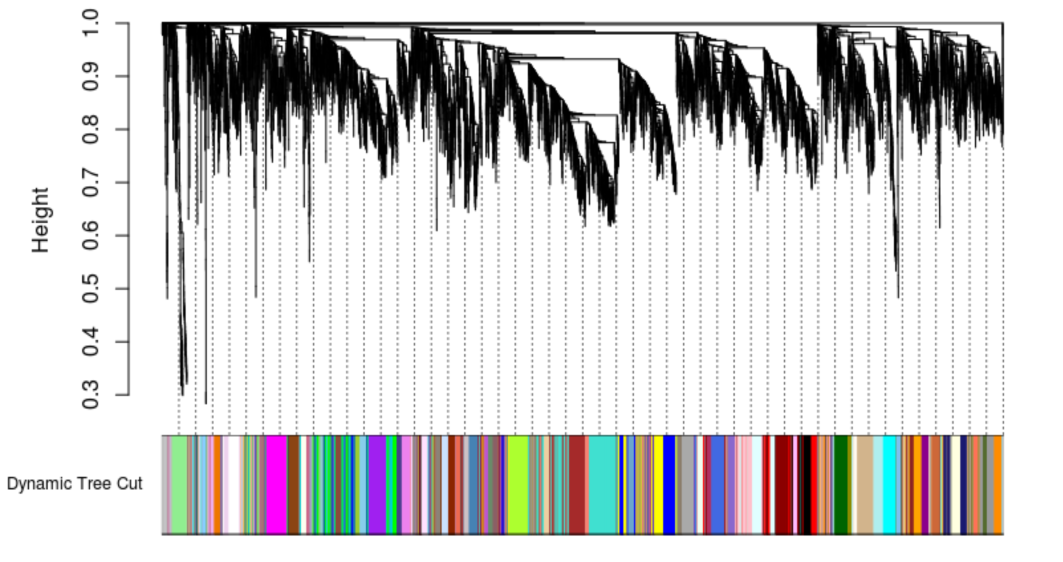

Supplement: Supplementary file 9 — Weighted gene co-expression network analysis of antioxidants-associated genes. Hierarchical cluster tree showing the modules of co-expressed genes. Each associated gene is represented by a leaf in the tree, while each module by a major tree branch. The lower panel in the figure shows the modules with assigned colours. (TIF 1403 kb) [file 12864_2019_5428_MOESM9_ESM.tif]

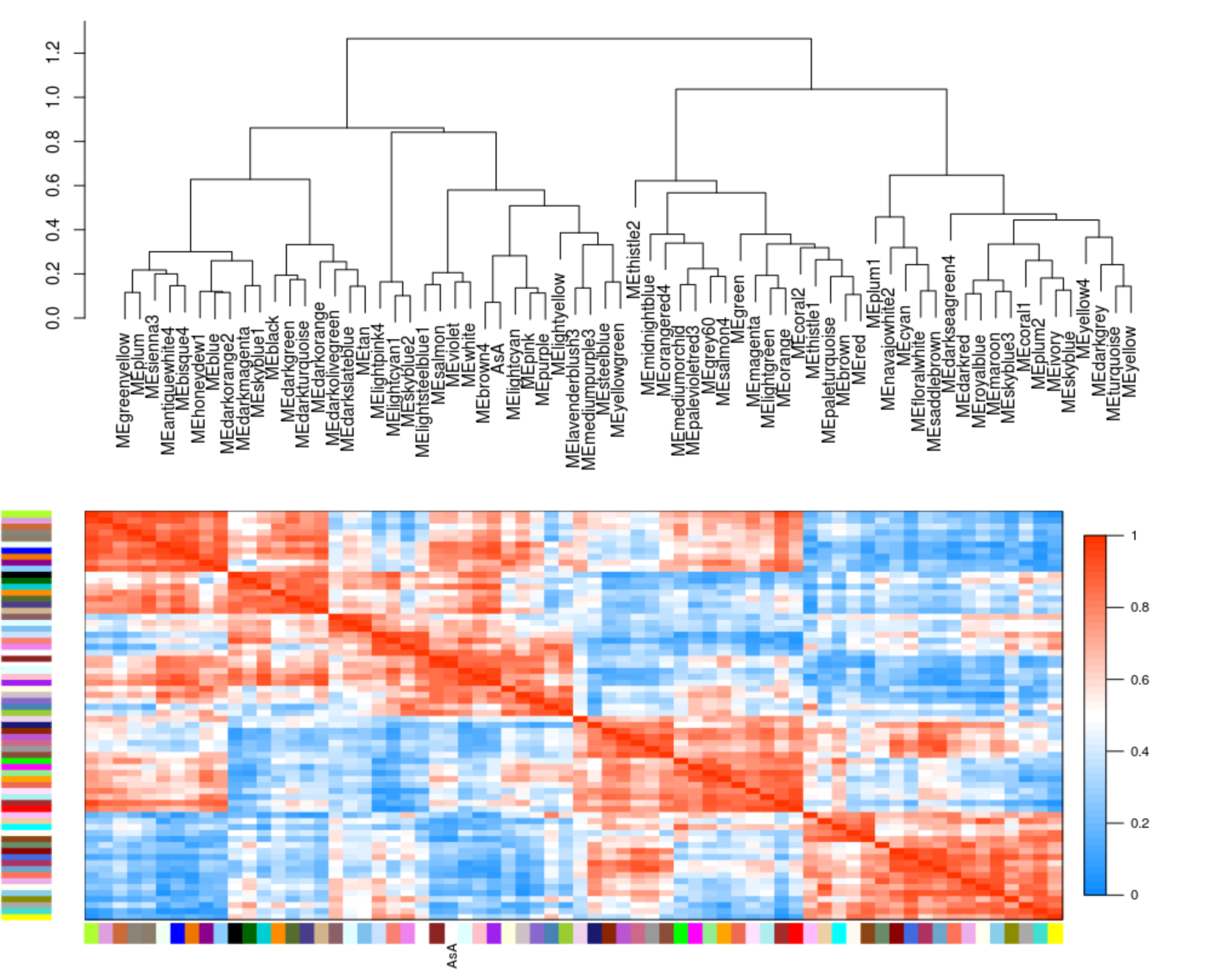

Supplement: Supplementary file 11 — Visualization of the eigengene network representing the relationships among modules and Ascorbic Acid (AsA) trait. Upper panel: hierarchical clustering dendrogram of the eigengenes. Lower panel: heatmap showing eigengenes adjacency. (TIF 2370 kb) [file 12864_2019_5428_MOESM11_ESM.tif]

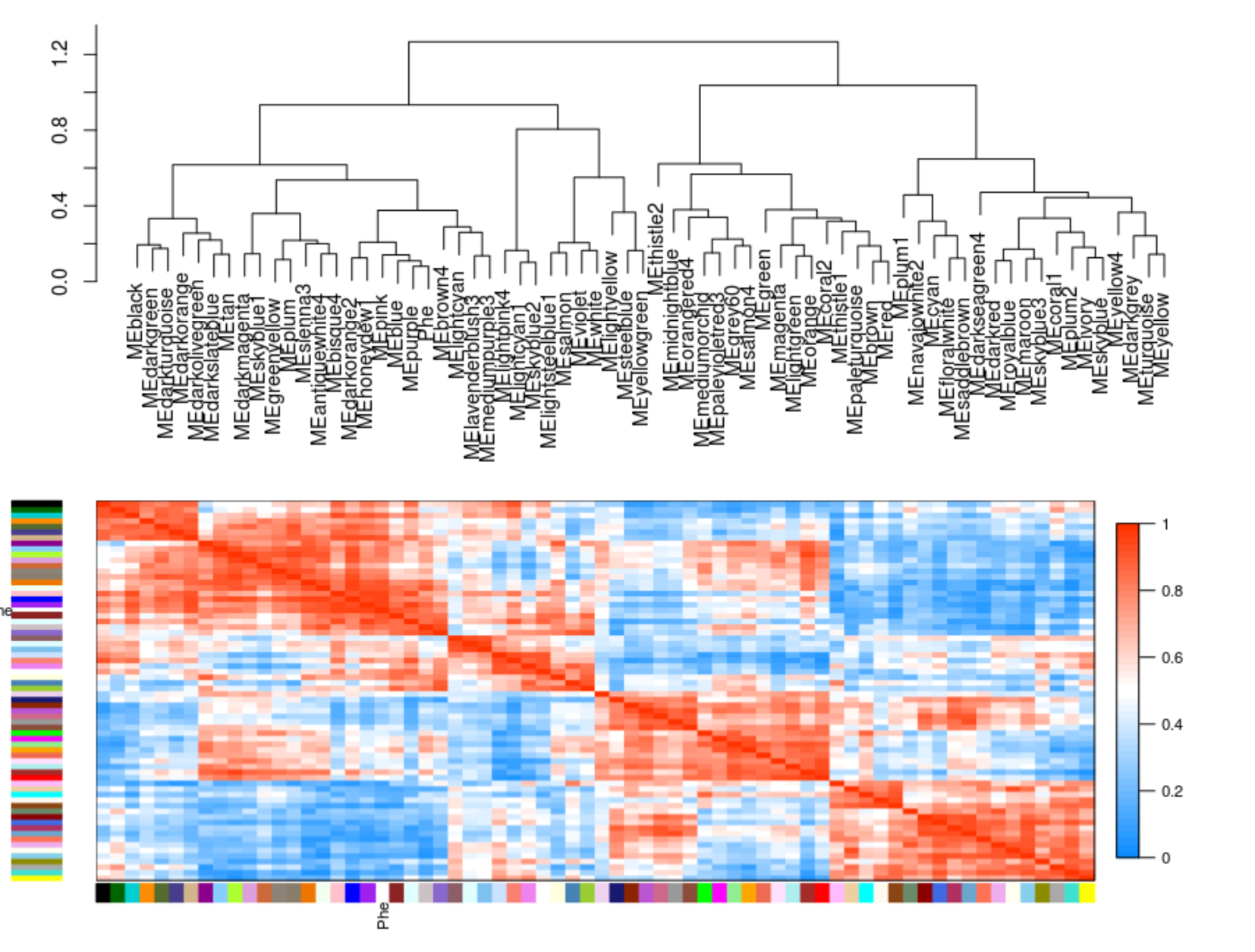

Supplement: Supplementary file 12 — Visualization of the eigengene network representing the relationships among modules and Phenolics (Phe) trait. Upper panel: hierarchical clustering dendrogram of the eigengenes. Lower panel: heatmap showing eigengenes adjacency. (TIF 2398 kb) [file 12864_2019_5428_MOESM12_ESM.tif]
