# Supplementary material for: New insights in the control of antioxidants accumulation in tomato by transcriptomic analyses of genotypes exhibiting contrasting levels of fruit metabolites
Source: BMC Genomics. 2019 Jan 15;20:43. doi: 10.1186/s12864-019-5428-4 (PMC6332538; doi:10.1186/s12864-019-5428-4)
Supplement: Supplementary file 14 — Table showing in each module identified by WGNA analysis the list of transcription factors and antioxidants-related genes. (DOCX 74 kb) [file 12864_2019_5428_MOESM14_ESM.docx]

**Additional File 14.** Table showing in each module identified by WGNA analysis the list of transcription factors and antioxidants-related genes.

| **Module Color** | **TF (description and Solyc ID)** | **Antioxidant-related gene (description and Solyc ID)** |
| --- | --- | --- |
| brown4  (AsA) | B3 (Solyc01g108140) | Ascorbate Peroxidase (Solyc02g083620) |
|  | FAR 1(Solyc01g112320) |  |
|  | Zinc Finger (Solyc10g080260) |  |
|  | Zinc Finger (Solyc01g087170) |  |
| Pink  (AsA and Phe) | GRAS (Solyc06g076290) | Galactokinase (Solyc01g058390) |
|  | Zinc Finger (Solyc10g079120) | Pectinesterase (Solyc03g112970) |
|  | Zinc Finger (Solyc04g056320) | O-methyltransferase (Solyc06g064510) |
|  |  | Hydroxycinnamoyl CoA shikimate/quinate hydroxycinnamoyltransferase (Solyc07g005760) |
|  |  | Malonyl CoA anthocyanin 5-O-glucoside-6-O-malonyltransferase (Solyc10g008650) |
|  |  | Polygalacturonase A (Solyc10g080210) |
| Purple (AsA and Phe) | HD-ZIP (Solyc04g074700) | UDP-glucosyltransferase (Solyc01g107850) |
|  | HB-other (Solyc08g061140) | Polygalacturonase (Solyc07g042160) |
|  | MADS-box (Solyc07g052700) | Ascorbate peroxidase (Solyc09g007270) |
|  | - | Laccase 22/L-ascorbate oxidase (Solyc07g052230) |
| Blue  (Phe) | bHLH (Solyc12g100140) | UDP-glucosyltransferase (Solyc01g107830) |
|  | CONSTANS (Solyc12g096500) | UDP-glucosyltransferase (Solyc09g092500) |
|  | GRAS (Solyc07g052960) | Anthocyanidin 3-O-glucosyltransferase (Solyc04g010110), |
|  | ERF4 (Solyc12g008350) | Cinnamoyl-CoA reductase-like protein (Solyc08g005120), |
|  | NAC/NOR (Solyc10g006880) | Hydroxycinnamoyl-CoA shikimate/quinate hydroxycinnamoyl transferase (Solyc02g079490) |
|  | HD-ZIP (Solyc02g063520) | Caffeoyl-CoA O-methyltransferase 1 (Solyc08g006830) |
|  | FUL1 (Solyc06g069430) | 4-coumarate-coa ligase (Solyc11g069050) |
|  | HY5 (Solyc08g061130) |  |
|  | bHLH (Solyc10g079050) |  |
|  | HSF A3 (Solyc09g065660) |  |
|  | Histone H1 (Solyc03g121840) |  |
|  | bHLH (Solyc07g043580) |  |
|  | C2H2L (Solyc10g084180) |  |
|  | BZIP (Solyc06g049040) |  |
|  | APRR2 (Solyc08g077230) |  |
|  | LOB (Solyc02g092550) |  |
|  | ERF12 (Solyc02g077840) |  |
|  | SCL (Solyc06g036170) |  |
|  | HSF A3 (Solyc06g053960) |  |
|  | BZIP (Solyc01g104650) |  |
|  | HSF (Solyc02g090820) |  |
|  | NAC4 (Solyc11g017470) |  |
|  | BZIP (Solyc04g080740) |  |
|  | TERF 5 (Solyc09g007990) |  |
|  | PHE1 (Solyc01g102260) |  |
|  | ARR11 (Solyc06g061030) |  |
|  | BLH1 (Solyc06g074120) |  |
|  | ARF 9 (Solyc08g008380) |  |
|  | AP2A (Solyc03g044300) |  |
|  | NFYC-2 (Solyc01g079870) |  |
|  | B3 (Solyc12g007300) |  |
